# ATPase copper transporting beta contributes to cisplatin resistance as a regulatory factor of extracellular vesicles in head and neck squamous cell carcinoma

**DOI:** 10.1038/s41417-025-00975-9

**Published:** 2025-10-17

**Authors:** Tatsuo Ogawa, Kisho Ono, Shoji Ryumon, Hotaka Kawai, Kohei Sato, Koki Umemori, Kunihiro Yoshida, Kyoichi Obata, Yuki Kunisada, Tatsuo Okui, Kuniaki Okamoto, Hitoshi Nagatsuka, Fatemeh Momen-Heravi, Soichiro Ibaragi

**Affiliations:** 1 Department of Oral and Maxillofacial Surgery, Faculty of Medicine, Dentistry and Pharmaceutical Sciences, Okayama University, Okayama, Japan.; 2 Department of Orofacial Sciences, School of Dentistry, University of California San Francisco, San Francisco, CA, USA.; 3 Cancer Biology and Immunology Laboratory, School of Dentistry, University of California San Francisco, San Francisco, CA, USA.; 4 Department of Oral Pathology and Medicine, Faculty of Medicine, Dentistry and Pharmaceutical Sciences, Okayama University, Okayama, Japan.; 5 Department of Maxillofacial Diagnostic and Surgical Science, Field of Oral and Maxillofacial Rehabilitation, Graduate School of Medical and Dental Sciences, Kagoshima University, Kagoshima, Japan.; 6 Department of Dental Pharmacology, Faculty of Medicine, Dentistry and Pharmaceutical Sciences, Okayama University, Okayama, Japan.

## Abstract

Cisplatin (CDDP) resistance remains a major clinical challenge in the treatment of head and neck squamous cell carcinoma (HNSC). Our group identified ATPase copper transporting beta (ATP7B) as a mediator of CDDP resistance through its role in drug efflux and small extracellular vesicle (sEV) secretion. Herein, we uncovered a novel mechanism by which ATP7B regulates sEV dynamics and the intercellular transmission of CDDP resistance. Using transcriptomic analyses of HNSC datasets, we demonstrate that ATP7B expression correlates with endocytosis- and epithelial-mesenchymal transition (EMT)-related gene sets and with elevated levels of EV-associated proteins. CDDP-resistant HNSC cells exhibited upregulated ATP7B, Rab5/Rab7, and preferentially secreted HSP90- and EpCAM-rich sEVs. These sEVs were leading to increased ATP7B expression and reduced CDDP sensitivity in recipient cells. A pharmacological inhibition of sEV biogenesis with GW4869 suppressed ATP7B and Atox1 expressions, inhibited late endosome maturation, and significantly enhanced CDDP-induced apoptosis in HNSC cells. In vivo, GW4869 reduced the sEV protein content and ATP7B expression in xenograft tumors. These findings establish that ATP7B is a critical modulator of sEV cargo and resistance propagation. Our results highlight a previously unrecognized ATP7B–sEV axis driving chemoresistance and identify sEV inhibition as a promising strategy to overcome therapeutic failure in HNSC.

## Graphical Abstract

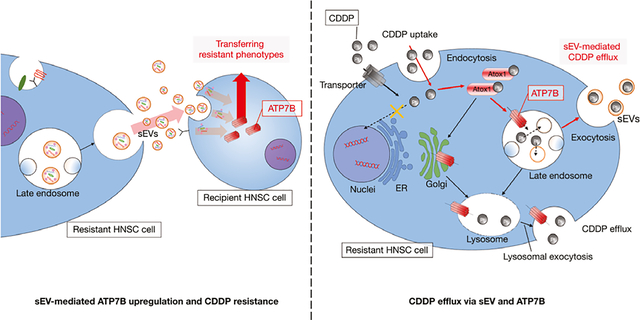

## INTRODUCTION

The chemotherapy drug cisplatin (CDDP) is widely used to treat head and neck squamous cell carcinoma (HNSC), but its efficacy is often compromised by resistance. CDDP resistance is known to arise multifactorially through the complex involvement of multiple mechanisms, including abnormalities in drug uptake/excretion, DNA repair, apoptosis inhibition, and the roles of cancer stem cells and the tumor microenvironment [1–3]. Among these, drug efflux is a process that actively expels CDDP from cancer cells by using intracellular transporters, thereby reducing the effectiveness of CDDP [1]. CDDP is transported into cells via the copper transport proteins 1 and 2 (CTR1 and CTR2), adenosine triphosphate (ATP) ase copper transporting alpha and beta (ATP7A and ATP7B), multidrug resistance proteins (MRPs), and ATP-binding cassette transporters; an abnormal expression or localization changes in these transport proteins are one of the primary causes of CDDP resistance [4–7].

Our research group has focused on the increased expression and function of ATP7B as a pump that actively transports CDDP out of cells as a CDDP-resistance factor in HNSC [8, 9]. ATP7B protein, as a P-type ATPase, plays an important role in maintaining copper homeostasis within cells and promoting copper transport. When this ATPase regulatory function is impaired, Wilson’s disease develops, causing copper metabolism disorders [10, 11]. In contrast, ATP7B has been shown to promote the transport of platinum agents and to be involved in the development of resistance to platinum agents such as CDDP [12]. The identification of the factors that regulate the functions of ATP7B would be an important step toward solving the difficult problem of CDDP resistance in HNSC chemotherapy.

Small extracellular vesicles (sEVs) were recently observed to be a major regulatory factor in drug resistance in many types of cancer [13]. As vesicles surrounded by a lipid bilayer membrane that are released from many types of cells [14, 15], sEVs are present in all body fluids and are known to function as important mediators of intercellular communication by transmitting various signals derived from donor cells. Some sEV mechanisms that are involved in the development of chemotherapy resistance play an important role in the resistance to cancer drugs by mediating the communication between cancer cells and their environment and by transporting bioactive molecules that may promote resistance [13, 16]. In addition, sEVs may induce resistance by encapsulating genetic information, membrane-bound proteins, and/or drugs [13]. The mediators of resistance-conferring molecular transfer via sEVs include genetic materials such as micro (mi)RNA and long non-coding (lnc)RNA, as well as proteins [17–19]. In antibody therapy, sEVs bind to antibodies and may inhibit the antibodies’ delivery to cancer cells [20].

The ATP7B transport pathway as part of the extracellular excretion of CDDP is thought to be a secretory pathway involving various vesicular structures such as secretory vesicles, secretory lysosomes, and sEV formation via late endosomes [21]. However, the actual mechanism remains unclear. In addition to these mechanisms, it would be interesting to determine whether CDDP-resistant cells transfer ATP7B derived from sEVs to recipient cells, thereby altering the phenotype of the recipient cells; this has also not yet been reported [21].

One of our research group’s earlier studies demonstrated two phenomena: (i) the loss of ATP7B in HNSC cells by RNA interference (RNAi) suppressed the secretion of sEVs, and (ii) inhibitors of the generation of sEVs suppressed the expression of ATP7B [8]. Our in vitro experiments demonstrated that this interaction causes a CDDP synergistic effect [8]. We conducted the present study to elucidate the localization dynamics of the ATP7B involved in CDDP resistance and the role of sEVs as resistance factors. By comprehensively analyzing sEVs and cells with the use of a transcriptomic database, we sought to clarify the relationship between sEV-associated molecules and ATP7B in the acquisition of CDDP resistance, thereby exploring the potential of sEVs as new targets for diagnostic and therapeutic strategies against HNSC.

## MATERIALS AND METHODS

### Reagents

Novus Biologicals (Littleton, CO, USA) was the source of the antibodies against ATP7B (#NB100-360) used in the western blotting analysis and Atox1 (#NBP1-06611). Antibodies against ATP7B (#sc-373964) used in immunocytochemistry and CD9 (#sc-13118) were purchased from Santa Cruz Biotechnology (Dallas, TX). The antibody against CD63 (#EXOAB-CD63A-1) was purchased from System Biosciences (Palo Alto, CA). Antibodies against Rab5A (#2143S), Rab7 (#2094S), HSP90 (4874S), EpCAM (VU1D9, #2929S), cleaved caspase 3 (5A1E), caspase 3 (D3R6Y), E-cadherin (24E10, #3195S) and vimentin (D21H3, #5741S) were purchased from Cell Signaling Technology (Danvers, MA). The antibody against GM130 (EP892Y, #ab52649) and horseradish peroxidase (HRP)-conjugated anti-β-actin antibody (#ab49900) were purchased from Abcam (Cambridge, MA). Anti-rabbit IgG AlexaFluor488 (#A-11008), anti-rabbit IgG AlexaFluor568 (#A-11077), and anti-mouse IgG AlexaFluor488 (#A-11001) were purchased from ThermoFisher Scientific (Waltham, MA).

Cisplatin (Randa^®^ Injection) was obtained from Nippon Kayaku (Tokyo). GW4869, a selective inhibitor of sphingomyelinase, was obtained from Cayman Chemicals (Ann Arbor, MI) and suspended in DMSO solvent as an EV-secretion inhibitor before use.

### Cell culture

The human head and neck cancer cell lines SAS, HSC-3, HSC-4, and OSC-19 were obtained from the Japanese Collection of Research Bioresources (JCRB) Cell Bank (Osaka, Japan) and cultured in Dulbecco’s modified eagle’s medium containing 10% fetal bovine serum (FBS) in a humidified incubator at 37 °C and 5% CO_2_ as described [22]. Cells were recently authenticated by short tandem repeat profiling and confirmed to be free of mycoplasma contamination before use.

### Public data collection and analysis

The *ATP7B, EPCAM* and *HSP90AA1* expression data of 564 samples (44 normal samples and 520 tumor samples) of the patients with HNSC in The Cancer Genome Atlas Program (TCGA)-HNSC database were downloaded based on the University of California, Santa Cruz (UCSC) Xena database (https://xenabrowser.net/datapages/). The datasets GSE202030 and GSE115119 include RNA-sequence (RNA-seq) data from parent cell lines and CDDP-resistant sublines of a total of three types of HNSC cells, which were downloaded from the GEO database (https://www.ncbi.nlm.nih.gov/geo/).

Dataset GSE65858 includes microarray data from 270 HNSC clinical species, and dataset GSE159067 includes RNA-seq data from 102 HNSC clinical samples, which were also downloaded from the GEO database. We divided the 507 HNSC patients registered in TCGA into a high-expression group (*n* = 127) and a low/medium-expression group (*n* = 380) based on ATP7 mRNA expression levels, and into a co-high-expression group (as *EPCAM*^High^*HSP90AA1*^High^; *n* = 167) and other group (*n* = 340) based on EPCAM and HSP90AA1 mRNA expression levels. We obtained the Kaplan–Meier plot of the patients’ overall survival.

### Enrichment analysis of ATP7B protein-related networks

We examined the protein-protein interaction (PPI) network of ATP7B by using the search tool for the Retrieval of Interacting Genes Database (STRING v.12.0; https://string-db.org/). The ATP7B-related proteins in the PPI databases (*n* = 11) were further characterized by gene ontology (GO) and pathway analyses using the ShinyGO database. GO terms were obtained for biological process (BP), cellular components (CC), molecular functions (MF), and pathway enrichment with the use of the Kyoto Encyclopedia of Genes and Genomes (KEGG). The top five results for the most significant terms by false discovery rate (FDR) values were selected for visualization. The 11 ATP7B-related proteins were compared between samples based on RNA-seq data from the datasets GEO202030 and GEO115119, which were visualized as heat maps.

### Extraction of gene data derived from HNSC samples and cell lines, and the gene set enrichment analysis

We conducted the gene set enrichment analysis (GSEA) by using RNA-seq data of HNSC clinical samples in dataset GSE159067 and cell lines in the Genomics of Drug Sensitivity in Cancer (GDSC) database. For clinical samples, based on the gene information in dataset GSE159067, including 102 HNSC clinical samples, we extracted 40 samples with high *ATP7B* expression and 40 samples with low *ATP7B* expression. For cell lines, based on the half-maximal inhibitory concentration (IC50) values for CDDP of 40 HNSC cell lines in the GDSC database, we divided CDDP responders and non-responders using the overall median IC50 value as the cutoff point. The enrichment score was defined as the significance level at an FDR < 25%. Hallmark and KEGG gene sets were obtained from the MSigDB network (https://www.gsea-msigdb.org/).

### LinkedOmics database analysis and subcellular localization evaluation

As a multiomics data analysis platform that includes data from all 32 TCGA cancer types and 10 Clinical Proteomic Tumor Analysis Consortium (CPTAC) cancer cohorts, the LinkedOmics database (http://www.linkedomics.org/login.php) provides the opportunity to explore and visualize gene expression profiles. Using LinkedOmics, we identified co-expressed proteins of ATP7B in normal and tumor tissues of the CPTAC-HNSC patient cohort by obtaining Pearson’s correlation coefficients, and the results are displayed as heat maps and volcano maps. We used the ShinyGO (GO-CC) and Enricher databases (http://maayanlab.cloud/enricher/) (Jensen’s compartment) to evaluate the subcellular localization of the top 50 protein species that were positively correlated with ATP7B.

### The CDDP-resistance model

We developed the CDDP-resistance model by growing the HNSC cell lines SAS and HSC-3 in increasing sublethal concentrations of CDDP in the growth medium as described previously [8]. The starting dose of CDDP was ~1 μg/mL, applied for 72 h. The medium was then replaced to let the cells recover for a further 72 h. Thereafter, the CDDP concentrations were augmented by 0.5 μg/mL every 72 h. The medium was changed every 3 days and maintained at the established CDDP concentration. The final CDDP concentration was set at a maximum of 3 μg/mL. This development phase was conducted for ~6 months, after which the IC50 was re-assessed. The resistant cell lines obtained (SAS-R and HSC-3-R) were cultivated under the same conditions as their parental cell lines.

### Cell viability assay

The resistance of HNSC cells to CDDP was evaluated at the IC50. The cell viability used to determine the IC50 value was measured by an MTT assay kit (Cayman Chemicals, Ann Arbor, MI, USA). In brief, HNSC cells were further cultured and then treated with 10 μL of MTT. After incubation for 3 h, the medium was removed and 100 μL of DMSO was added. The optical density at 570 nm was measured, and the cell survival rate in each group was calculated. The IC50 of the HNSC cells to CDDP was then obtained using relative survival curves.

The cell viability assay was performed as described previously [8]. CDDP was added to the HNSC cells at the IC50 for 24 h after cell seeding. Phosphate-buffered saline (PBS) was added to the control group as a substitute for CDDP. Cells were detached or disassembled using Trypsin/EDTA at 24 h after the addition of CDDP or PBS, and the number of cells was counted using a Countess^®^ Automated Cell Counter (Thermo Fisher Scientific, Waltham, MA). Cell viability was determined by converting the ratio of the number of cells in the CDDP-added group to that in the PBS-added group.

### Invasion assay

The invasion assay was conducted with BioCoat Matrigel invasion chambers (#354480, Corning, Corning, NY). Cells grown in a serum-free culture medium were applied to the upper chamber. In the lower chamber, a medium containing 10% FBS as a chemoattractant was applied. After incubation for 24 h at 37 °C, the remaining cells were removed with a cell scraper. The filters were then fixed with 10% paraformaldehyde for 10 min and washed with PBS three times, and the cell nuclei were stained with the 4’,6-diamidino-2-phenylindole (DAPI) (#ab104139, Abcam, Cambridge, MA) for 5 min, followed by a washing step with PBS. Cell counting was performed in four random microscope fields per well.

### Whole cell lysate

The whole cell lysate (WCL) was prepared as described previously [8, 14, 15, 23]. Cells cultured in a 10-cm dish were lysed in 200 μL/dish of RIPA buffer (1% NP-40, 0.1% SDS, 0.5% deoxycholate, and EDTA-free protease inhibitor cocktail in PBS) and collected by a cell scraper. The cells were further lysed using a 25-gauge syringe for 10 strokes and then incubated for 30 min on ice. Spheroids were treated with ultrasonic crushing. The protein concentrations were analyzed using a BCA protein assay (Thermo Fisher Scientific).

### sEV fractions

sEV fractions were prepared from the serum-free culture supernatants at 48 h after medium replacement using a modified polymer-based precipitation method. The WCL was prepared at the same time as described above. Briefly, the cell culture supernatant was centrifuged at 2000 × *g* for 30 min at 4 °C and then centrifuged at 10,000 × *g* for 30 min at 4 °C. The supernatants were filtered with a 0.2-μm pore filter. The pass-through was concentrated using an ultrafiltration device for molecular weight 100 K to collect the sEV fraction.

The concentrate was applied to polymers of Total Exosome Isolation (Thermo Fisher Scientific). The sEV fractions were suspended in 100–200 μL of PBS without calcium or magnesium. For the protein assay, 10 μL of 10× RIPA buffer and 10 μL of 100× protease inhibitor cocktail (Sigma, St. Louis, MO) were added to 100 μL of the sEV fraction and incubated on ice for 15 min. Protein concentrations were analyzed with a micro-BCA protein assay (Thermo Fisher Scientific).

sEVs derived from mouse serum were collected as follows. Albumin was removed using Minute Albumin Depletion Reagent (#WA-01, Invent Biotechnologies, Plymouth, MN) according to the manufacturer’s guidelines. Serum samples were centrifuged at 3000 × *g* for 30 min. sEVs were isolated using a Total Exosome Isolation Reagent (from serum) (Thermo Fisher Scientific) according to the manufacturer’s guidelines. Specifically, 100 μL of serum underwent mixing with 20 μL of reagent following a 5:1 ratio. Following a 24-h incubation at 4 °C, the solution underwent centrifugation at 10,000 × *g* for 10 min at 4 °C. The resulting sEV pellet was then suspended in 100 μL of PBS without calcium or magnesium for subsequent analyses.

### Western blotting

Equal amounts of the WCL or sEV lysate were subjected to sodium dodecyl sulfate polyacrylamide gel electrophoresis (SDS-PAGE) and then transferred to a polyvinylidene difluoride membrane using a semi-dry method. Membranes were blocked in 5% skim milk in Tris-buffered saline containing 0.05% Tween-20 for 60 min, incubated with primary antibodies (dilution 1:1000), and then incubated with HRP-conjugated secondary antibodies (dilution 1:5000). Blots were visualized using a Clarity ECL substrate and a ChemiDoc MP system (Bio-Rad, Hercules, CA). We loaded the same protein amount per lane in each Western blotting experiment. Quantitative protein band intensities were quantified using ImageJ software (ver. 1.53K; U.S. National Institutes of Health, Bethesda, MD).

### Immunocytochemistry

An immunocytochemistry (ICC) analysis was performed as follows. Cells were fixed with 4% paraformaldehyde (PFA) for 20 min and then permeabilized with 0.1% Triton-X100 for 10 min. The fixed cells were treated with methanol containing 0.3% H_2_O_2_ and blocked within 3% bovine serum albumin in PBS containing 0.1% Tween-20 for 30 min at room temperature (RT). Slides were incubated with anti-ATP7B (dilution 1:200), anti-GM130 (dilution 1:100), anti-Atox1 (dilution 1:200), anti-cleaved caspase 3 (dilution 1:200), anti-E-cadherin (dilution 1:1000), or anti-vimentin (dilution 1:100) antibodies overnight at 4 °C and with anti-rabbit IgG AlexaFluor488 (dilution 1:500), anti-rabbit IgG AlexaFluor568 (dilution 1:1000), and/or anti-mouse IgG AlexaFluor488 (dilution 1:1000) for 60 min. Slides were stained with nuclear stain DAPI. Fluorescence images were taken using a BZ-X700 microscope (Keyence, Osaka, Japan). Quantitative fluorescence intensities were quantified using ImageJ software (vU.S. National Institutes of Health).

### Transmission electron microscopy

Transmission electron microscopy (TEM) imaging was carried out as follows. A 400-mesh copper grid coated with formvar/carbon films was treated hydrophilically. The sEV suspension (5–10 μL) was placed on Parafilm^®^, and the grid was floated on the sEV liquid and left for 15 min. The sample was negatively stained with 2% uranyl acetate solution for 2 min. Extracellular vesicles on the grid were visualized with 20,000× magnification with an H-7650 transmission electron microscope (Hitachi, Tokyo).

### Particle size distribution

A part of the sEV fraction was diluted with PBS to a volume up to 40 μL and then analyzed using a Zetasizer Nano ZSP (Malvern Panalytical, Malvern, UK) in a range of diameters from 0.3 to 10,000 nm, as described [8, 14, 15].

### Fluorescence-labeled sEV transmission

An ExoGlow-protein EV labeling kit (System Biosciences, Palo Alto, CA) was used for sEV labeling per the manufacturer’s protocol. Briefly, isolated sEVs were incubated for 20 min with the labeling dye (1:500 in PBS) at 37 °C to induce vesicular protein conjugation with the dye molecules. The solution was then treated with ExoQuick-TC solution and incubated for 2 h at 4 °C, followed by centrifugation at 10,000 × *g* for 10 min to remove unlabeled reagent molecules. For a negative control, sterile PBS was incubated with labeling dye and treated in the same manner. Labeled sEV pellets were dissolved in PBS. The labeled sEVs were added to the culture medium of SAS cells and HSC-3 cells in 96-well plates at 20 μg/mL. The cells were fixed with 4% paraformaldehyde in PBS for 10 min and permeabilized with 0.5% Tween-20 in PBS for 5 min. The cells were incubated with ActinGreen488 (ThermoFisher) for 30 min and with DAPI for 5 min. Fluorescence images were taken using a BZ-X700 microscope (Keyence, Osaka, Japan).

### Wound healing assay

A total of 1.0 × 10^4^ cells/well were seeded in six-well plates. When the cells reached 90% confluence, an artificial wound was made across the monolayer using the tip of a 200-μL pipette. The cells were then washed with PBS and cultured in a serum-free medium 12 h. The wound width was measured microscopically at 0, 6, and 12 h.

### Three-dimensional (3D) spheroid culture

For the 3D spheroid culture, 1.0 × 10^5^ cells were seeded into each well of a 96-well ultra-low attachment (ULA) plate (#7007, Corning). Spheroid images were captured with a bright-field microscope (IX81; Olympus, Tokyo).

### Hypoxia detection assay of spheroids

The hypoxia conditions of the spheroids were measured using a hypoxia probe solution (LOX-1; MBL, Nagoya, Japan). Fluorescence images and the fluorescence intensities of the spheroids were obtained with the BZ-X700 microscope. Quantitative hypoxic intensities were quantified using ImageJ software (U.S. National Institutes of Health).

### Animal experiment

Female nude mice (BALB/c-nu/nu mice, 6–8 weeks old) were purchased from Shimizu Laboratory Suppliers (Kyoto, Japan) and were housed under pathogen-free conditions. SAS cells, 1 × 10^6^ cells per xenograft, were transplanted subcutaneously into the backs of BALB/c-nu/nu nude mice. After 7 days, the mice were divided into two groups: a control group and a GW4869 group (*n* = 4 per group). GW4869 (2.5 μg/g ≒ 50 μg/mouse) was administered intraperitoneally to mice 4×/week for 14 days. Control mice received xenografts as described above, followed by intraperitoneal DMSO. The mice were sacrificed at 21 days after transplantation under isoflurane anesthesia. No formal statistical sample size calculation was performed. The number of animals used was determined based on previous studies, common practice in xenograft tumor models, and the principle of minimizing animal use while ensuring reproducibility. We randomly divided mice of the same age and gender into two groups, one as the control group and the other as the experimental group. When assessed the outcome, authors who were not involved in the experiments were asked to blindly evaluate the results.

Total blood was obtained by cardiac puncture, and the serum was processed, isolated, and stored at −80 °C. The largest tumor diameter (*L*) and smallest tumor diameter (W) were measured, and the tumor volume (*V*) was calculated (*V* = *L* × *W*^2^). Tumors along with surrounding tissues were excised, immersion-fixed in 10% neutral buffered formalin, dehydrated, embedded in paraffin, and sectioned to prepare slides. The slides were used for immunohistochemistry (IHC).

### Immunohistochemistry

The immunohistochemistry (IHC) examination was performed using the anti-ATP7B antibody. The sections were deparaffinized in xylene for 15 min and rehydrated in graded ethanol solutions. Endogenous peroxidase activity was blocked by incubating the sections in 0.3% H_2_O_2_ in methanol for 30 min. The antigen in the sections was retrieved, and sections were blocked with 10% normal serum for 15 min. Next, the sections were incubated with primary antibodies at 4 °C overnight. Signal enhancement was performed by the avidin-biotin complex method (Vector Lab, Newark, CA). DAB (Histofine DAB substrate) was used for color development, and Myer’s hematoxylin was used for the counterstaining. The staining results were detected with an optical microscope (BX53, Olympus). The comparison of ATP7B-positive cells in the tumors was performed with ImageJ software (U.S. National Institutes of Health).

### Statistical analyses

Statistical analyses were conducted using GraphPad Prism. The differences between pairs of groups were analyzed by Welch’s *t*-test or Mann–Whitney U tests, and those among three or more groups were analyzed by a oneway or two-way analysis of variance (ANOVA) followed by Tukey’s or uncorrected Fisher’s post-tests, respectively. All tests were performed as two-tailed tests. A significance threshold of 0.05 was selected for statistical testing, and significance was defined as follows: *p < 0.05, **p < 0.01, ***p < 0.001, ****p < 0.0001. Data are expressed as the mean ± standard deviation (SD) unless otherwise specified.

## RESULTS

### The copper transport pathway network surrounding ATP7B contributes to CDDP resistance

**Fig. 1:**
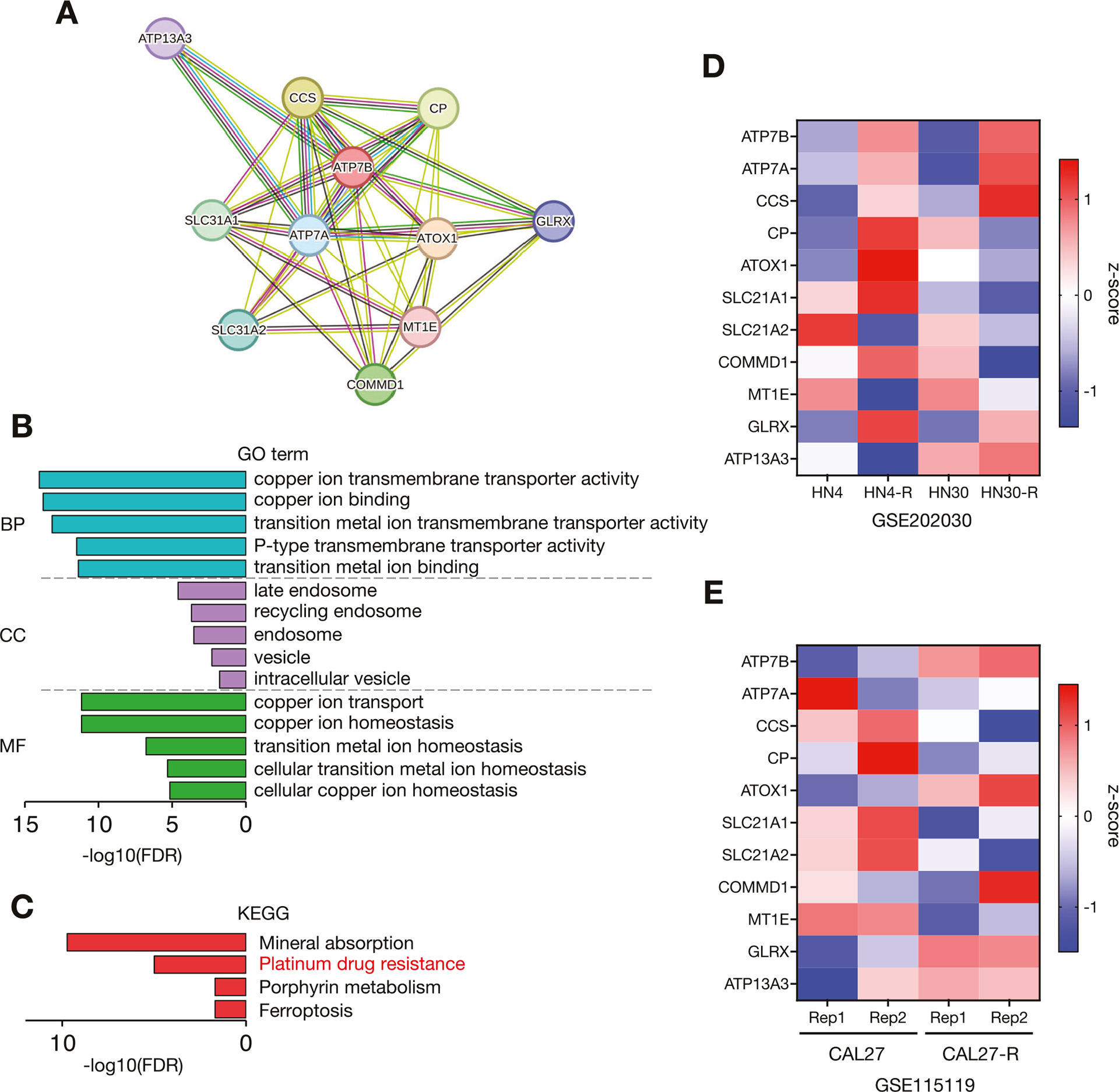
The results of the enrichment analysis of the ATP7B-rerlated proteins. **A** The protein-protein interaction (PPI) network among ATP7B-related proteins analyzed using the STRING database. **B** The gene ontology (GO) analysis of the 11 ATP7B-related proteins detected in the PPI database. The top five enriched categories for Biological Process (BP), Cellular Component (CC) and Molecular Function (MF) terms were analyzed using the ShinyGo database. **C** The KEGG pathway analysis of the 11 ATP7B-related proteins detected with the PPI databases. The KEGG pathway terms were analyzed using the ShinyGo database. Heat maps showing relative the gene expression comparison of the 11 ATP7B-related proteins in the parental HNSC cell lines (HN4, HN30 and CAL27) and their CDDP-resistant sublines (HN4-R, HN30-R, and CAL27-R) based on RNA-seq data from the GSE202030 (**D**) and GSE115119 (**E**) datasets. The GSE202030 dataset contained one sample for each cell line, and the GSE115119 dataset contained two replicates for each cell line.

Our group’s previous study demonstrated that the expression of ATP7B was elevated in CDDP-resistant HNSC cells, but it remained unclear how ATP7B-related proteins interacted with each other and in what biological processes, subcellular localization, and signaling pathways they were involved [8]. We thus accessed the STRING database of known and predicted PPIs to investigate which proteins are related to ATP7B physically and functionally. We identified 11 ATP7B-related proteins, including ATP7A and Atox1 (Fig. 1A).

To further explore the potential biological functions of these 11 ATP7B-related proteins, we performed gene ontology (GO) and KEGG enrichment analyses (Fig. 1C, D). As shown in Fig. 1B, C and listed in Supplementary Table S1, the results revealed that cooper ion transport was highly detected in the KEGG term categories biological process (BP), late endosome and recycling endosome in cellular components (CC), copper ion binding and cooper ion transmembrane transporter activity in molecular functions (MFs), and platinum drug resistance. Since the ATP7B-related proteins were enriched in terms of platinum drug resistance, we used the RNA-sequence data of several HNSC cell lines and their CDDP-resistant sublines in the GEO202030 and GEO115119 datasets to compare the relative expressions of the 11 ATP7B-related genes (Fig. 1D, E). In the GSE202030 dataset, *ATP7B*, *ATP7A*, *CCS*, and *GLRX* were commonly upregulated in CDDP-resistant sublines (HN4-R and HN30-R) of HN4 and HN30 cell lines, respectively (Fig. 1D). In the GSE115119 dataset, *ATP7B*, *ATOX1*, and *GLRX* were commonly upregulated in CDDP-resistant subline (CAL27-R) of CAL27 cell line (Fig. 1E). Taken together, the heat maps showed that in these two datasets, *ATP7B*, *ATP7A*, *ATOX1*, *CCS*, and *GLRX* were highly expressed in two or more of the three cell lines in CDDP-resistant sublines compared to their parentals (Fig. 1D, E).

These results suggested that the copper transport pathway network surrounding ATP7B is localized in endosomes and that the overexpression of ATP7B contributes to CDDP resistance.

### Endocytosis and epithelial mesenchymal transition are involved in the CDDP resistance induced by ATP7B upregulation

**Fig. 2:**
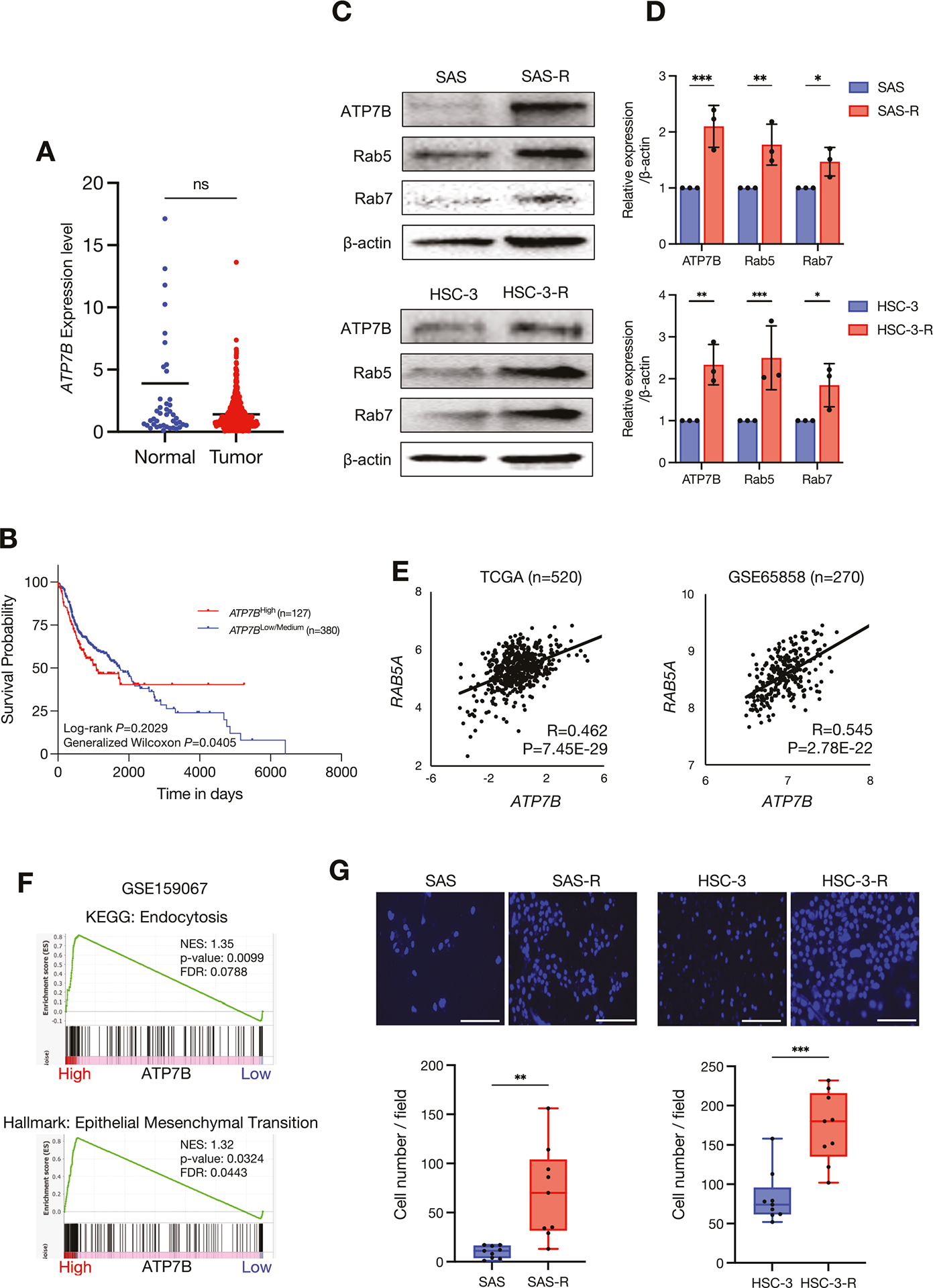
ATP7B as a poor prognostic factor and a CDDP-resistance factor in HNSC. **A** Scatterplot comparing the ATP7B expression in normal (*n* = 44) and tumor tissues (*n* = 520) from the TCGA-HNSC cohort. The lines indicate the mean value. Mann–Whitney test was used for pairwise comparison. ns: not significant. **B** Kaplan–Meier survival curves comparing the ATP7B high-expression group (*n* = 130) and a low/medium-expression group (*n* = 389) in the TCGA-HNSC cohort. The *p*-values correspond to those of the log-rank test and generalized Wilcoxon test comparing the survival curves. **C** Western blotting showing the comparisons of ATP7B, Rab5, and Rab7 in the parent and resistant cells of each cell line. β-Actin was used as the loading control. **D** The comparison of ATP7B, Rab5 and Rab7 expression levels in the parent and resistant cells of each cell line. The relative band intensities of the western blotting were normalized to β-actin by using Image J software (*n* = 3). Each bar indicates the mean. Error bars: standard deviation (SD). Two-way ANOVA with uncorrected Fisher’s LSD test was used for the comparison. *p < 0.05, **p < 0.01, ***p < 0.001. **E** Scatterplots for the correlation between the expressions of *ATP7B* and *RAB5A* in the HNSC clinical samples based on the TCGA-HNSC cohort (*left*, *n* = 520) and the GSE65858 dataset (*right*, *n* = 270). **F** The gene set enrichment analysis (GSEA) results showing the KEGG and hallmark signatures from the molecular signals database MSigDB by comparing 40 ATP7B high-expression and 40 low-expression groups of HNSC samples. Sets of genes related to endocytosis and epithelial-mesenchymal transition were significantly enriched in the ATP7B-high HNSC samples in the GSE159067 dataset. FDR: false discovery rate, NES: normalization enrichment score. **G** Representative images and the results of the comparison of the number of Matrigel invasion cells indicated by DAPI positivity after 24 h. DAPI-stained cells from each sample were counted in nine random fields. The lines indicate the mean and range. Welch’s *t* test was used for the comparison. **p < 0.01, ***p < 0.001.

To verify the clinical significance of *ATP7B* expression in HNSC, we investigated the differences in *ATP7B* expression between normal and tumor tissues from The Cancer Genome Atlas Program (TCGA)-HNSC cohort; no significant differences were revealed (Fig. 2A). However, the Kaplan–Meier plot showed that a high expression of *ATP7B* decreased the survival rate of HNSC patients (Fig. 2B).

To examine the relationship between CDDP resistance and ATP7B, we used the same resistant sublines of SAS (SAS-R) and HSC-3 (HSC-3-R) cells that were established in our earlier investigation [8]. There was no significant difference in cell proliferation between the parentals and the CDDP-resistant cells in the SAS or HSC-3 cell lines (Supplementary Fig. S1A). ATP7B was highly expressed in the SAS-R and HSC-3-R cells compared to their respective parentals (Fig. 2C, D).

Rab5 and Rab7, which are members of the Rab family of proteins, are localized in the endosome, and they deeply involved in endocytosis and the endosome maturation pathway [24]. To investigate the correlation between ATP7B and Rab5 and that between ATP7B and Rab7, we performed western blotting, which revealed a high expression of Rab5 and Rab7 in the CDDP-resistant cell lines, similar to ATP7B (Fig. 2C, D). We further used two large datasets that registered the transcriptomic gene profiles of HNSC clinical samples, and we observed that *ATP7B* and *RAB5A* showed significant positive correlations in both the TCGA-HNSC cohort and the GSE65858 dataset (Fig. 2E). The correlation between *ATP7B* and *RAB7A* was not clear (Supplementary Fig. S1B).

Next, to explore the key characteristics and pathways affected by *ATP7B* upregulation in HNSC, we performed a GSEA by comparing *ATP7B*-high and *ATP7B*-low HNSC clinical samples in the GSE159067 dataset. The GSEA results demonstrated that gene sets of endocytosis in the KEGG database were significantly enriched in the *ATP7B*-high HNSC samples (Fig. 2F). This was consistent with our findings of ATP7B, Rab5 and Rab7 upregulation in CDDP-resistant cells and the positive correlation between *RAB5A* and *ATP7B* in both HNSC cohorts (Fig. 2C, E). The GSEA results further showed that the gene set of epithelial-mesenchymal transition (EMT) was significantly enriched in the *ATP7B*-high HNSC samples (Fig. 2F). The other GSEA analysis comparing CDDP responders and non-responders in the HNSC cell lines obtained the same result (Supplementary Fig. S1C).

We investigated whether the acquisition of ATP7B upregulation-induced CDDP resistance affects EMT-related cell motility, and we observed that SAS-R and HSC-3-R had high invasiveness compared to their parentals (Fig. 2G). We also performed a wound healing assay, which revealed that the SAS-R subline exhibited increased migration compared to the SAS cell line (Supplementary Fig. S1D). Based on these findings, we speculate that the enhancement of this motility/invasive ability was associated with a decrease in E-cadherin and an increase in vimentin in the SAS-R cells (Supplementary Fig. S1E, F).

Together, the above-described results suggest that enhanced endocytosis and EMT function may be involved in the CDDP resistance caused by ATP7B upregulation in HNSC.

### High expression of ATP7B in HNSC upregulates sEV-associated proteins

**Fig. 3:**
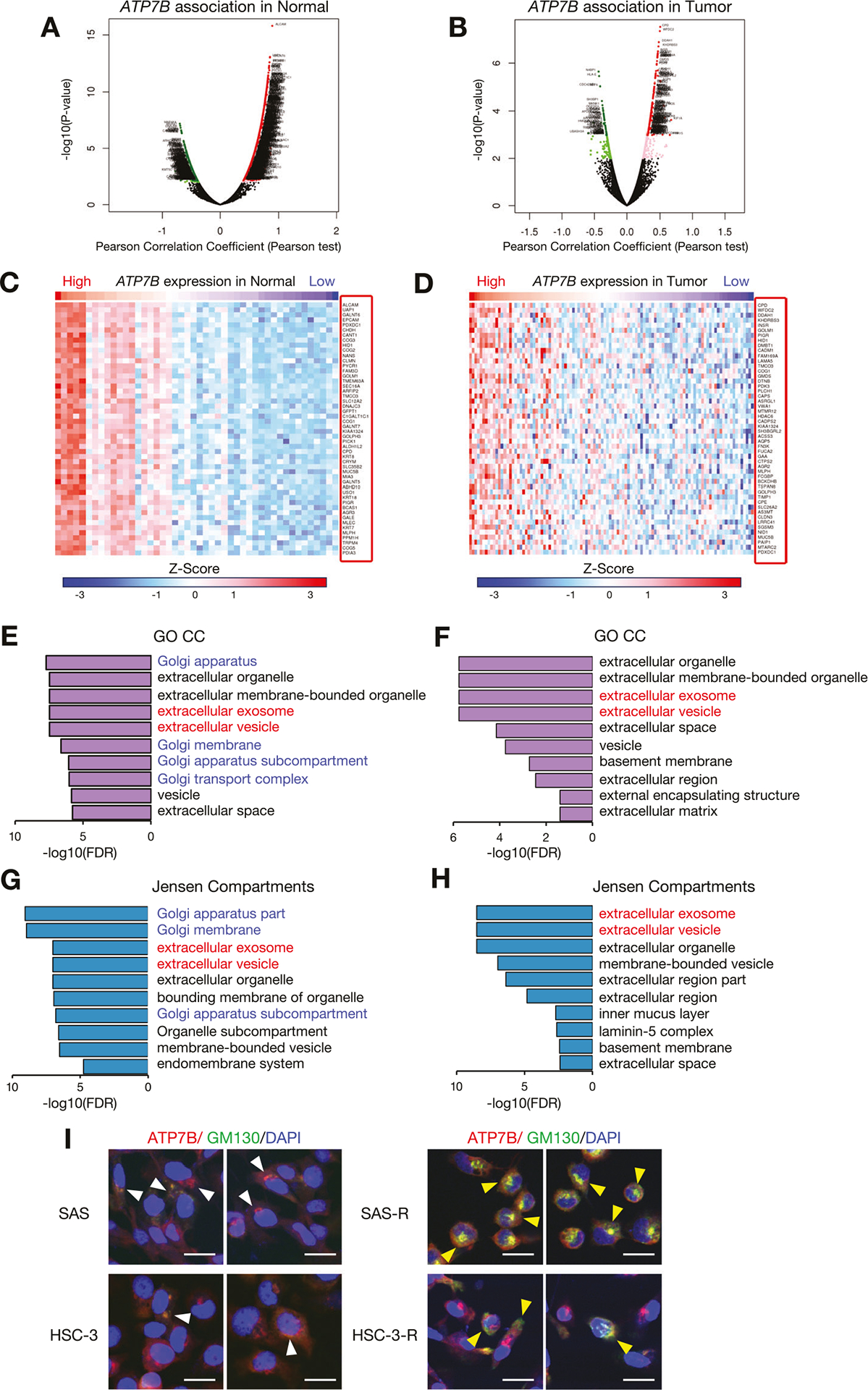
Proteins that are positively correlated with ATP7B expression and their subcellular localization in HNSC. Volcano maps of the proteins that were highly correlated with ATP7B according to Pearson’s test in the **A** normal tissues (*dark red dots*) and **B** tumor tissues (*dark red dots:* significant positive correlation) and (*dark green dots:* negative correlation, FDR < 0.05) in the CPTAC-HNSC cohort. Heatmaps showing the top 50 proteins that were positively correlated with ATP7B in the normal tissues (**C**) and tumor tissues (**D**) in the CPTAC-HNSC cohort. The *red frame* lists the protein names. The results of the GO analysis of the top 50 proteins that were positively correlated with ATP7B in the normal tissues (**E**) and tumor tissues (**F**). We analyzed the top 10 enriched categories for Cellular Components (CC) terms by using the shinyGo database. The subcellular localization evaluation of the top 50 proteins that were positively correlated with ATP7B in the normal tissues (**G**) and tumor tissues (**H**) based on the Jensen Compartments database. The Enricher database was used to analyze the top 10 enriched categories. **I** Immunocytochemistry (ICC) showing the ATP7B and GM130 protein expression in the parent and CDDP-resistant SAS and HSC-3 cells. ATP7B are shown in *red*, GM130 in *green* and DAPI in *blue*. The white arrowheads indicate the regions where ATP7B and GM130 are colocalize stained in the parent cells, while the yellow arrowheads indicate the regions where GM130 is singly stained in the CDDP-resistant cells. Scale bar: 20 μm.

To comprehensively evaluate how the ATP7B-correlated protein profile in HNSC contributes to CDDP resistance, we used the LinkedOmics database to examine ATP7B co-expressed proteins in normal and tumor tissues from the CPTAC-HNSC cohort. As shown in the volcano plots in Fig. 3A, B, a total of 1002 proteins had a significant positive correlation with ATP7B and 502 proteins were significant negatively related in the normal tissues (Fig. 3A), and 171 proteins had a significant positive correlation and 54 proteins were significant negatively related in the tumor tissues (FDR < 0.05) (Fig. 3B). Heatmaps of the top 50 proteins that were positively correlated with ATP7B in normal tissues (Fig. 3C) and tumor tissues (Fig. 3D) were also created.

To identify the cell structures and localizations in which these top 50 protein species are involved, we performed an enrichment analysis using two types of intra- or extracellular localization evaluation: GO-CC and Jensen compartment database (Fig. 3E–H, Supplementary Tables S2, S3). The analyses of both GO-CC and the Jensen compartment database identified “extracellular exosome” and “extracellular vesicle” as top terms in both the normal tissue (Fig. 3E, G, Supplementary Table S2) and tumor tissue (Fig. 3F, H, Supplementary Table S3). Another noteworthy point is that terms related to the Golgi apparatus were enriched in the normal tissue (Fig. 3E, G, Supplementary Table S2) but not identified in tumor tissue (Fig. 3F, H, Supplementary Table S3). ATP7B is known to be localized mainly in late endosomes, but since it functions to transport copper from the cytosol to the trans-Golgi network (TGN) under normal conditions, it should also be observed in the TGN [25]. However, in some diseases, it has been reported that the ATP7B localization in the TGN disappears depending on pathological conditions [25]. We examined the correlation between *ATP7B* expression and various TGN markers in normal and tumor tissues. In the normal tissues, TGN markers showed a significantly strong positive correlation with *ATP7B* expression, whereas in the tumor tissues, the correlation was clearly attenuated (Supplementary Fig. S2). We further performed double staining of GM130, which is one of the TGN marker, and ATP7B to verify whether the ATP7B localization changes also affect CDDP resistance. Immunocytochemistry (ICC) staining showed that ATP7B and GM130 showed colocalization in both parent SAS and HSC-3 cells, whereas in their respective CDDP-resistant cells, ATP7B localization shifted and single intracellular staining sites for GM130 became prominent (Fig. 3I). These results led us to speculate that (i) a high expression of ATP7B in HNSC may be related to the sEV-related events, (ii) the correlation between ATP7B and the TGN may be weakened in HNSC tumors, and (iii) intracellular localization change of ATP7B from the TGN may be involved in CDDP resistance.

### CDDP-resistant sEVs increase ATP7B expression and confer CDDP resistance in recipient cells

**Fig. 4:**
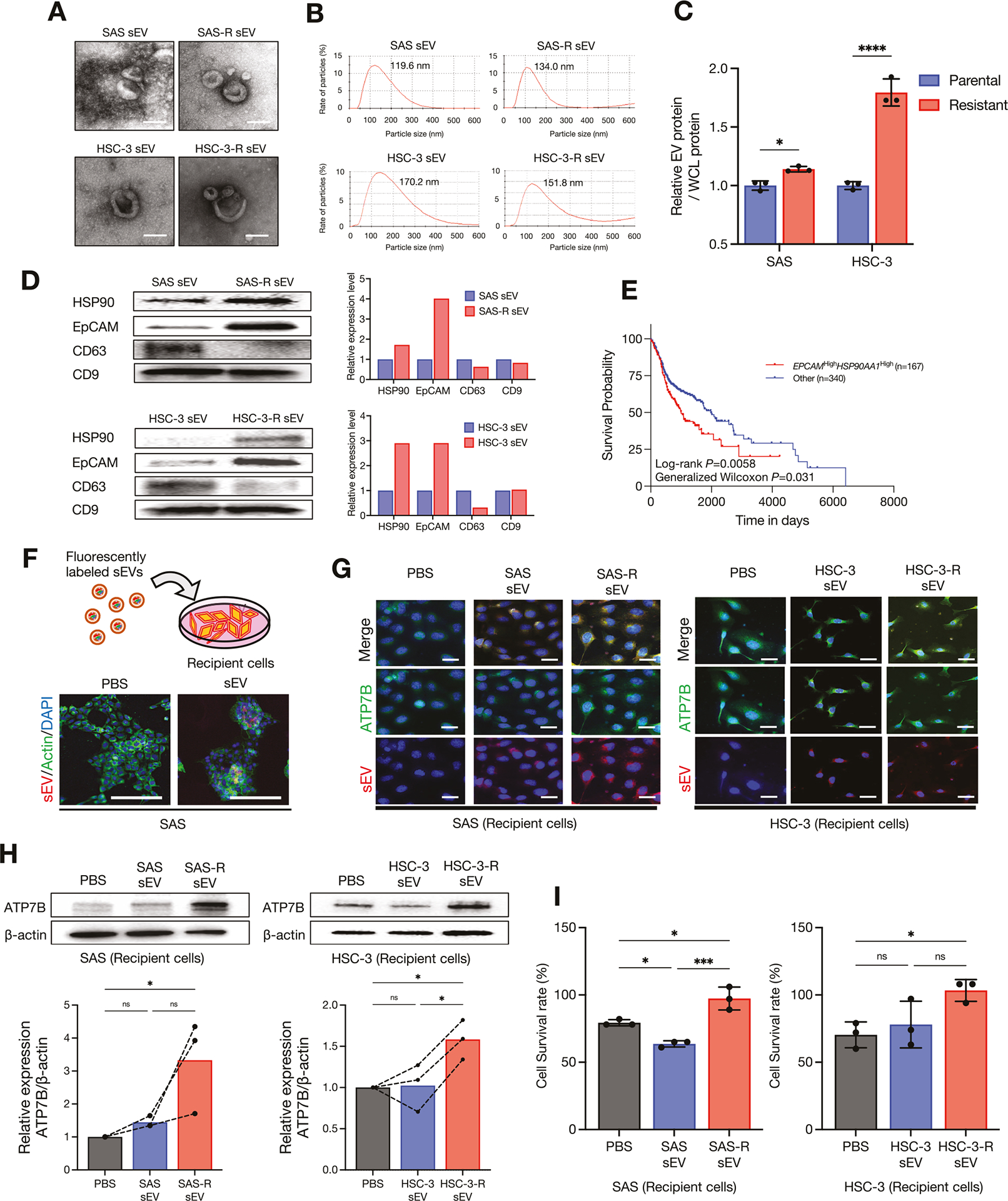
Enhanced ATP7B and drug resistance by CDDP-resistant HNSC-derived sEV transmission. **A** Representative transmission electron microscopy (TEM) images of sEVs derived from each cell lines. Scale bar: 100 nm. **B** Representative particle diameter distribution of sEVs derived from each cell line. Peak values were 100–200 nm. **C** The relative sEV protein per cellular lysate protein in the parent and resistant cells of each cell line (*n* = 3 technical replications). Each bar indicates the mean. Error bars: SD. Two-way ANOVA with uncorrected Fisher’s LSD test was used for the comparison. *p < 0.05, ****p < 0.0001. **D** Western blots for the comparison of HSP90, EpCAM, and tetraspanins (CD9, CD63) in sEVs derived from the parentals and resistant cell lines. The relative HSP90, EpCAM, and tetraspanins band intensities of the western blotting were measured by using Image J software. **E** Kaplan–Meier survival curves comparing the *EPCAM*^High^*HSP90AA1*^High^ expression group (*n* = 167) and the other group (*n* = 340) in the TCGA-HNSC cohort. The *p*-values correspond to those of the log-rank test comparing the survival curves. **F** A simple schema of sEV uptake into culturing recipient cells and representative images of fluorescently labeled sEV transmission. sEV is shown in *red*, actin in *green*, and DAPI in *blue*. EV-free phosphate-buffered saline (PBS) was used as a control. Scale bar: 200 μm. **G** ICC showing the ATP7B protein expression and fluorescently labeled sEVs after the transmission of each sEV into recipient cells for 24 h. Each parent cell line was used as recipient cells. ATP7B is shown in *green*, sEV in *red*, and DAPI in *blue*. Scale bar: 100 μm. **H** Western blots for the comparison of the ATP7B expression after the transmission of each sEV into recipient cells for 24 h. EV-free PBS was used as a control, and each parental cell line was used as the recipient cells. β-Actin = loading control. The relative ATP7B band intensities of the western blotting were normalized to β-Actin by using Image J software (*n* = 3). Each bar indicates the mean. The line graphs represent corresponding independent technical replicate. One-way ANOVA with Tukey’s multiple comparison test was used for the comparison. ns: not significant, *p < 0.05. **I** The CDDP sensitivity in recipient cells after the transmission of each sEV for 24 h, expressed as the cell survival rate (*n* = 3). Each bar indicates the mean. Error bars: SD. One-way ANOVA with Tukey’s multiple comparison test was used for the comparison. ns: not significant, *p < 0.05, ***p < 0.001.

The database analysis revealed an association between ATP7B and the sEVs (Fig. 3). Our group’s earlier investigation revealed that loss of ATP7B suppressed the sEV secretion from HNSC cells and reduced the expression of CD9, EpCAM, and HSP90 in sEVs [8]. In the present study, we investigated the relationship between ATP7B and sEVs by analyzing sEVs secreted from HNSC cell lines and their CDDP-resistant sublines. As depicted in Fig. 4A, B, the sEV fractions isolated from the parental and CDDP-resistant cells showed no obvious change in the sEV morphology or size. Both SAS-R and HSC-3-R cells showed an increase in sEV secretion compared to their respective parental cells, with HSC-3-R showing particularly significant increases (Fig. 4C).

Notably, both the SAS-R- and HSC-3-R-derived sEVs showed high HSP90 and EpCAM expression and low CD63 expression compared to their respective parental sEVs (Fig. 4D), demonstrating that the contents of the sEV cargo had changed. Incidentally, the differences in the protein expression within sEVs varied depending on the source of the HNSC cell lines (Supplementary Fig. S3A). Interestingly, the Kaplan–Meier plot showed that a co-high expression of *EPCAM* and *HSP90AA1*, genes encoding EpCAM and HSP90 highly expressed in the CDDP-resistant sEVs, significantly decreased the survival rate of HNSC patients (Fig. 4E). For an investigation of how the changes in the sEV cargo that are associated with CDDP resistance affect recipient cells, each sEV was transmitted to its parental cells (Fig. 4F). The fluorescently labeled sEVs tended to be taken up more by the recipient cells in proportion to the amount of sEVs transmitted (Supplementary Fig. S3B). ICC staining showed that parental sEVs and CDDP-resistant sEVs were taken up into the recipient cells at similar levels, but the CDDP-resistant sEVs showed increased ATP7B expression in the recipient cells compared to the parental sEVs (Fig. 4G). Similarly, western blotting revealed a significant upregulation of ATP7B in the recipient cells by CDDP-resistant sEVs (Fig. 4H), and the CDDP-resistant sEVs significantly reduced the CDDP sensitivity of recipient cells compared to the parental sEVs (Fig. 4I). In summary, these results indicate that CDDP-resistant sEVs induced an upregulation of ATP7B in recipient cells via sEV cargo regulation, thereby promoting a reduction in CDDP sensitivity.

### The EV inhibitor GW4869 enhanced the cytotoxic effect of CDDP by inhibiting late endosomes

**Fig. 5:**
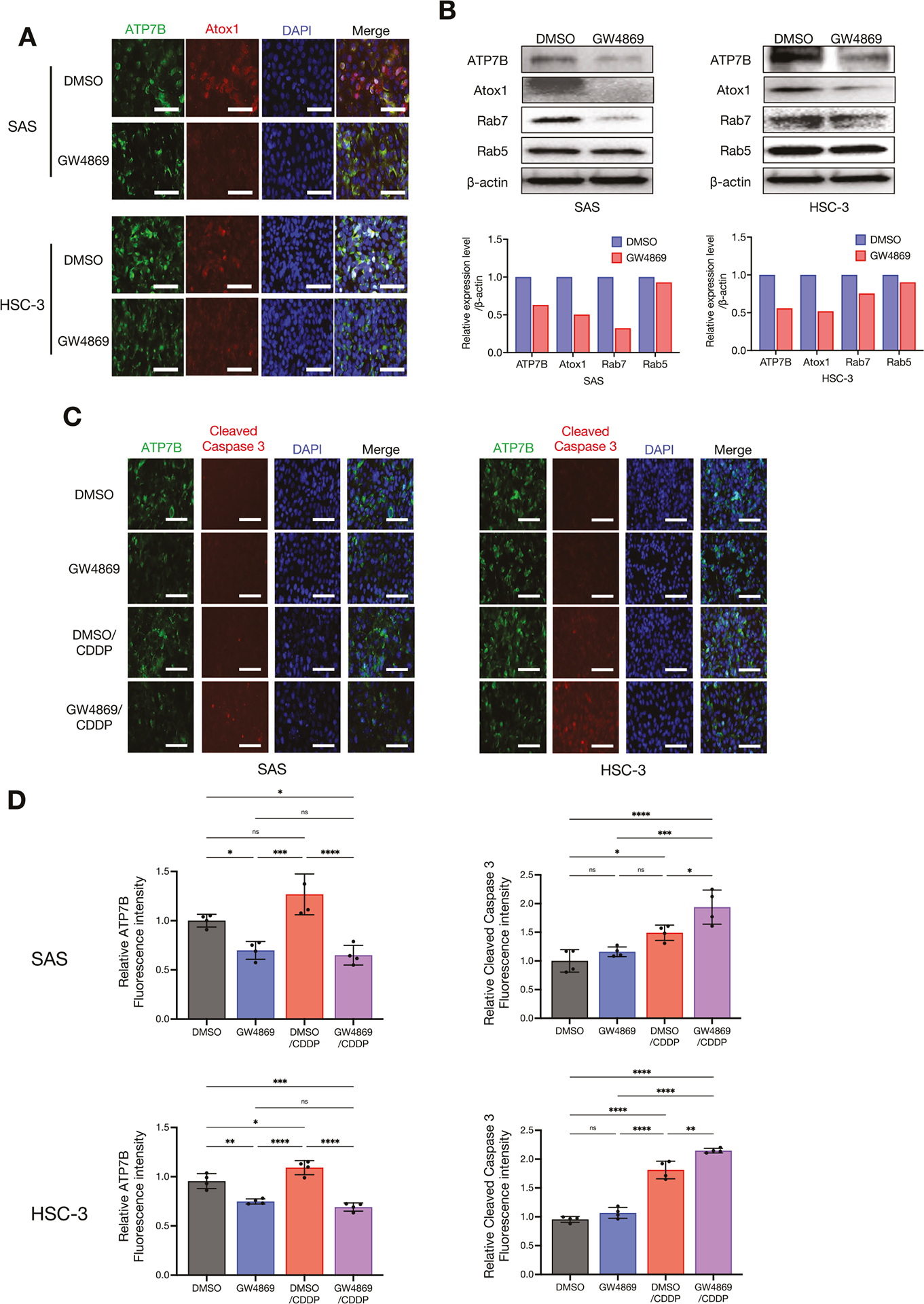
The suppression of intracellular ATP7B and the acquisition of a synergistic effect with CDDP by the EV inhibitor GW4869. **A** ICC showing the ATP7B and Atox1 protein expressions in the cell lines treated with or without GW4869 for 24 h. ATP7B is shown in *green*, Atox1 in *red*, and DAPI in *blue*. Scale bar: 50 μm. **B** Western blots showing the ATP7B, Atox1, Rab7, and Rab5 protein expressions in the cell lines treated with or without GW4869 for 24 h. β-Actin = loading control. The relative ATP7B, Atox1, Rab7, and Rab5 band intensities of the western blotting were normalized to β-actin by using Image J software. **C** ICC showing the ATP7B and cleaved caspase 3 protein expressions in each cell line treated with GW4869 and/or CDDP for 24 h. ATP7B: *green*, cleaved caspase 3: *red*, and DAPI: *blue*. Scale bar: 50 μm. **D** The comparison of ATP7B and cleaved caspase 3 expression levels in each cell line treated with or without GW4869 and/or CDDP. The upper row shows SAS, the lower row shows HSC-3, the left shows ATP7B, and the right shows cleaved caspase 3. Relative each ATP7B and cleaved caspase 3 fluorescent intensities of the ICC images were normalized to DAPI intensities using Image J software (*n* = 4). Each bar indicates the mean. Error bars: SD. One-way ANOVA with Tukey’s multiple comparison test was used for the comparison. ns: not significant, *p < 0.05, **p < 0.01, ***p < 0.001, ****p < 0.0001.

**Fig. 6:**
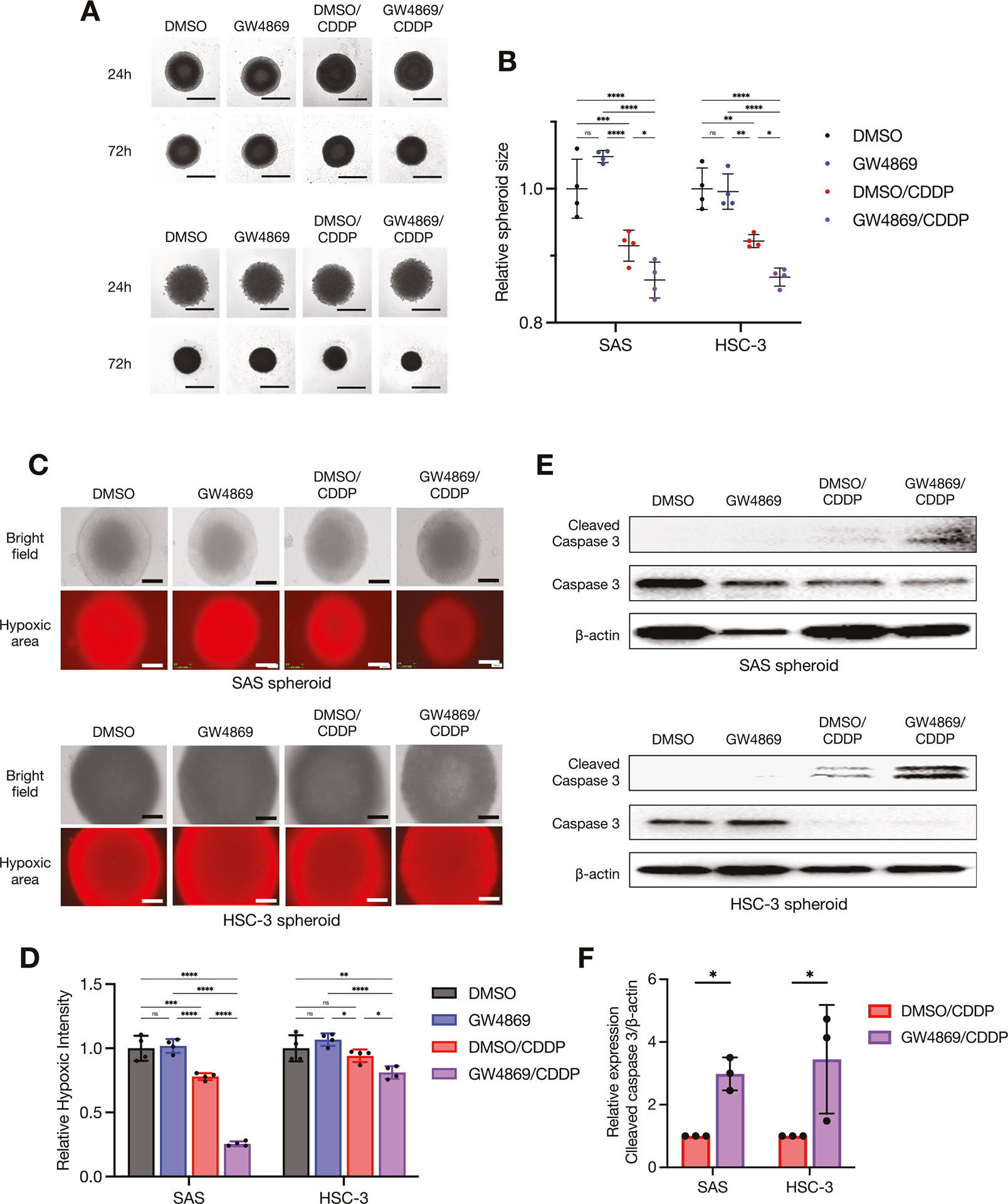
GW4869 enhanced the cytotoxic effect of CDDP in three-dimensional tumors in vitro. **A** Representative images of SAS and HSC-3 spheroids treated with GW4869 and/or CDDP for 24 h and 72 h. Scale bar: 1 mm. **B** The comparison of spheroid size in each cell line treated with or without GW4869 and/or CDDP in 72 h. Each relative spheroid size was normalized based on area (*n* = 4). Error bars: SD. Two-way ANOVA with Tukey’s multiple comparison test was used for the comparison. ns: not significant, *p < 0.05, **p < 0.01, ***p < 0.001, ****p < 0.0001. **C** Representative images of SAS and HSC-3 spheroids in bright fields and hypoxic areas. The intensity of the *red areas* indicates that the stronger the red signal, the stronger the hypoxia. Scale bar: 200 μm. **D** The comparison of hypoxic intensities in each cell line treated with or without GW4869 and/or CDDP. Relative each hypoxic intensities of the spheroid images were normalized using Image J software (*n* = 4). Each bar indicates the mean. Error bars: SD. Two-way ANOVA with Tukey’s multiple comparison test was used for the comparison. ns: not significant, *p < 0.05, **p < 0.01, ***p < 0.001, ****p < 0.0001. **E** Western blots showing the cleaved caspase 3 and caspase 3 protein expressions in spheroids of each cell line treated with or without GW4869 and/or CDDP for 24 h. β-Actin = loading control. **F** The comparison of cleaved caspase 3 expression level in spheroids of each cell line treated with CDDP-alone or CDDP+GW4869 combination for 24 h. The relative band intensities of the western blotting were normalized to β-actin by using Image J software (*n* = 3). Each bar indicates the mean. Error bars: SD. Two-way ANOVA with uncorrected Fisher’s LSD test was used for the comparison. *p < 0.05.

We continued the aim of our prior investigation [8] to evaluate the effects of the EV inhibitor GW4869 on CDDP treatment at the molecular level. GW4869 is a drug that inhibits the maturation process from early endosomes to late endosomes and is involved in the transport and degradation of substances within cells [26]. Hence, we also examined the effect on the expression of the copper metallochaperon protein Atox1, which is a chaperon that transports copper from the cytoplasm into the lumen of late endosomes via ATP7B [27], among the highly expressed molecules in the CDDP-resistant sublines shown in Fig. 1D, E. As shown in Fig. 5A, B, the treatment with GW4869 suppressed not only the ATP7B expression but also the Atox1 expression in HNSC cells. Interestingly, GW4869 suppressed Rab7 expression, but Rab5 expression was not affected (Fig. 5B). We further investigated how GW4869 contributes to the cytotoxic effects of CDDP and ATP7B expression. Our earlier study demonstrated that GW4869 alone did not cause cytotoxicity or a decrease in the survival rate in 2D-cultured HNSC cells, and that GW4869 potentiates the cytotoxic effect of CDDP when GW4869 and CDDP are used in combination [8]. In the present study, we assessed the reproducibility of the changes in ATP7B and cleaved caspase 3 by conducting an ICC examination of 2D-cultured HNSC cells treated with GW4869 and CDDP. The results revealed that the CDDP-induced enhancement of cleaved caspase 3 expression was also more pronounced in the CDDP+GW4869 combination group compared to the CDDP-alone group, and a tendency toward decreased ATP7B expression was observed in GW4869-treated group (Fig. 5C, D). We further applied this reproducibility to 3D-cultured HNSC cells, as spheroids (Fig. 6). GW4869 had no effect on spheroid growth based on size, whereas CDDP inhibited the growth, and this effect was even more pronounced with the CDDP+GW4869 combination (Fig. 6A, B). It was noteworthy that the CDDP+GW4869 combination resulted in a significant decrease in the hypoxic area compared to the CDDP-alone group (Fig. 6C, D). In addition, similar to the 2D-culture results, the enhancement of cleaved caspase 3 expression by CDDP was more pronounced in the CDDP+GW4869 combination group than in the CDDP-alone group in the 3D-culture (Fig. 6E, F).

**Fig. 7:**
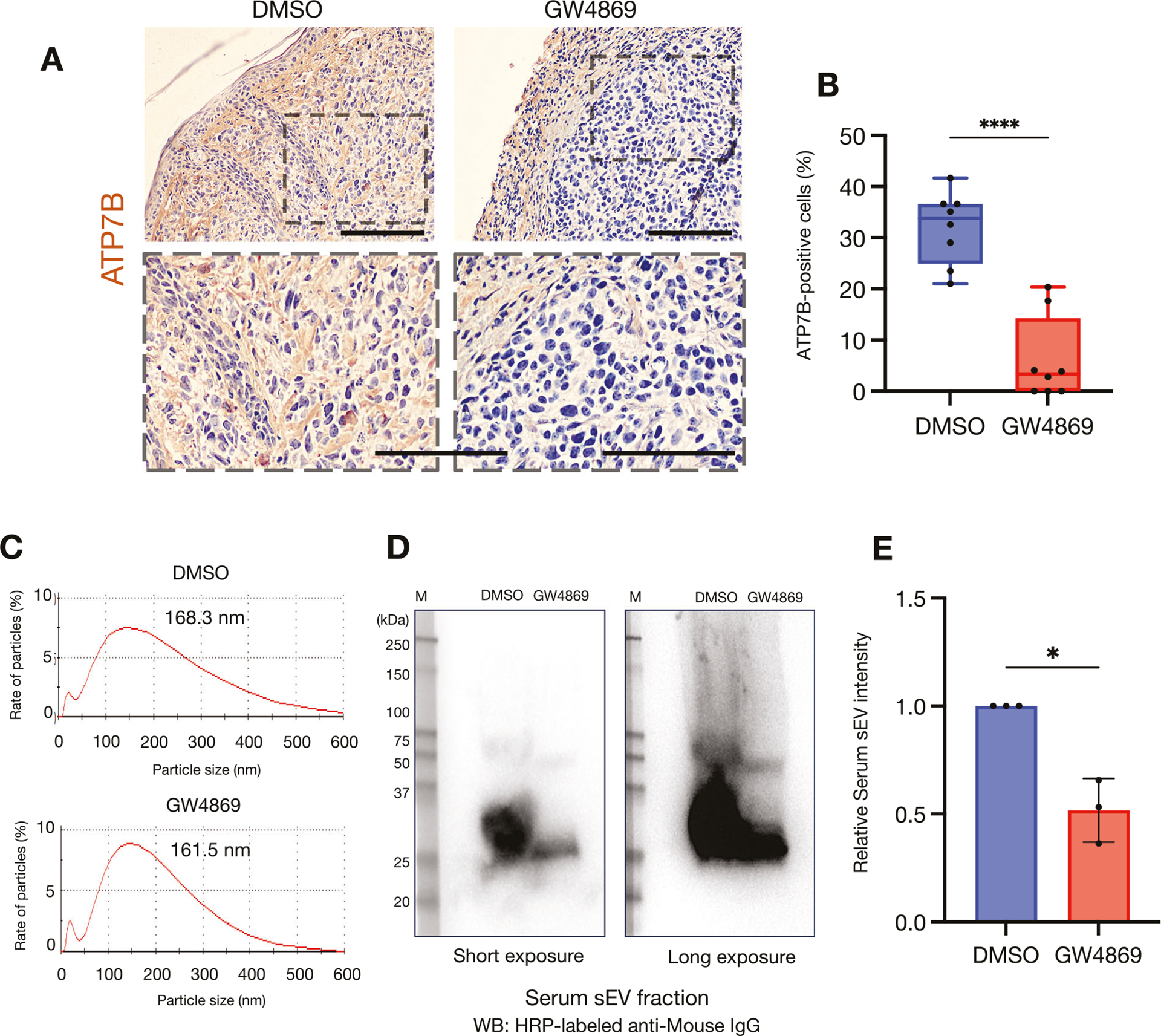
GW4869 suppressed the expression of ATP7B in the in vivo tumors. **A** Representative immunohistochemical staining images of ATP7B in excised tumor tissues treated with or without GW4869. *Upper row:* low-magnification images. *Lower row:* high-magnification images. The area enclosed by the *dotted line* at the top matches the image at the bottom. Scale bar: 100 μm. **B** The proportion of ATP7B-positive cells in tumor tissues treated with or without GW4869. ATP7B-positive cells were counted in two random fields per tumor, for a total of eight fields (*n* = 8). The lines indicate the mean and range. Welch’s *t* test was used for the comparison. ****p < 0.0001. **C** Representative particle diameter distribution of serum sEVs derived from mice treated with or without GW4869. Peak values were 100–200 nm. **D** Western blotting showing sEV proteins from mice treated with or without GW4869 using HRP-labeled anti-mouse IgG. The same amount of sample standardized by protein quantification was applied to each lane. **E** The comparison of serum sEV proteins levels from mice treated with or without GW4869. The relative serum sEV proteins band intensities of the western blotting were measured by using Image J software (*n* = 3). Each bar indicates the mean. Error bars: SD. Welch’s *t* test was used for the comparison. *p < 0.05.

We used a mouse xenograft model to examine the effects of GW4869 on biological tumors (Fig. 7). SAS cells transplanted subcutaneously into the back of mice formed tumors in the same area (Supplementary Fig. S4A). After the tumor formation, we continuously administered GW4869 to the mice intraperitoneally for 2 weeks, and the mice were then sacrificed and the tumors were resected (Supplementary Fig. S4A). There was no difference in the volume of the resected tumors between groups (Supplementary Fig. S4A, B). However, the immunohistochemical staining of the resected tumor specimens demonstrated that the expression of ATP7B was significantly increased in the GW4869-treated group compared to the control group (Fig. 7A, B, Supplementary Fig. S4C). We further isolated sEVs from the whole blood of mice in each group. The particle size of the isolated sEVs was ~160–170 nm in both groups (Fig. 7C). Interestingly, however, the western blotting of equal amounts of serum-derived sEV fractions standardized by protein concentration demonstrated a clear reduction in the expressions of sEV proteins following the GW4869 treatment (Fig. 7D, E).

Taking these new findings together with those of our earlier investigation [8] suggests that the inhibition of sEV generation and the suppression of ATP7B expression by GW4869 may enhance the therapeutic effect of CDDP (Figs. 5–7).

## DISCUSSION

Our group had revealed in 2024 that (i) CDDP-resistant HNSC cells highly expressed ATP7B and (ii) the loss of ATP7B increased the cells’ CDDP sensitivity [8]. Although several studies have suggested that ATP7B is a factor contributing to CDDP resistance, its localization remains controversial. ATP7B was reported to localize to late endosomes in many studies [28]. ATP7B is a P-type ATPase that is also present in the TGN under steady-state conditions, and it is involved in the maintenance if copper homeostasis within cells [29, 30]. ATP7B has been shown to colocalize with the TGN markers TGN38, p230, and syntaxin6 [31–33]. The results of our present PPI database analysis revealed that no molecules localized in the TGN were detected among ATP7B-related proteins, and many were localized to late endosomes and recycling endosomes (Fig. 1A, B) (Supplementary Table S1). However, the enrichment analysis of the CPTAC-HNSC cohort showed that Golgi-localized proteins positively correlated with ATP7B expression were upregulated in normal tissues but disappeared in tumor tissues (Fig. 3, Supplementary Fig. S2, Supplementary Tables S2, S3). In some diseases, it has been reported that Golgi-localized ATP7B disappears as the disease progresses, resulting in localization abnormalities and loss of function [25]. Changes in the localization of ATP7B may influence carcinogenesis and cancer progression.

In the present study, CDDP-resistant HNSC cells exhibited not only enhanced ATP7B expression but also enhanced expressions of endosome-localized proteins such as Rab5 and Rab7 (Fig. 2C). One of the main mechanisms of CDDP resistance is thought to be the capture and sequestration of CDDP within endosomes, which affects the intracellular CDDP distribution [34, 35]. Since the target site of CDDP is nuclear DNA, CDDP captured in endosomes cannot reach the target site [36]. Rab5 is localized in early endosomes and regulates the initial stages of endocytosis [24]. Rab7 is localized in late endosomes and controls the maturation of endosomes, their transition to lysosomes, and the degradation of substances in lysosomes [24]. Rab5 is replaced by Rab7 as endosomes mature through its own activation [24]. The enhancement of these two Rab proteins is associated with enhanced endocytosis, which supports the mechanism of endosome trapping as the basis of CDDP resistance (Fig. 2C).

It was also noteworthy that the ATP7B expression in the present HNSC clinical specimens showed a positive correlation with *RAB5A*, and that an endocytosis-related gene set was enriched in the HNSC cases in which ATP7B was highly expressed (Fig. 2D, E). However, no clear correlation with *RAB7A* was observed (Supplementary Fig. S1B). It has been shown that ATP7B is present in Rab7-positive late endosomes under high copper conditions [37], whereas another study showed that ATP7B translocation is independent of Rab7 [38]. No reports have described a correlation between ATP7B and Rab5 expression. An elucidation of the complex and intertwined scenario of copper transport pathways and Rab5,7-mediated endocytosis control mechanisms may provide a crucial clue for overcoming CDDP resistance.

In a continuation of our earlier study [8], we focused herein on the roles of ATP7B expression and sEV dynamics in CDDP resistance. The enrichment analysis of the CPTAC-HNSC cohort revealed that the proteins that were positively correlated with ATP7B were associated with sEV terms (Fig. 3, Supplementary Tables S2, S3). Another research group reported that EVs derived from drug-resistant cells contain ATP7A, ATP7B, and MPR-2 [39]. Our current verification did not confirm that ATP7B is abundant in resistant sEV (no data shown). However, the results of our analyses demonstrated that sEVs derived from CDDP-resistant cells possess HSP90- and EpCAM-rich characteristics (Fig. 4D). Our group has also shown that HSP90- and EpCAM-rich sEVs are preferentially secreted from highly metastatic oral cancers and promote cancer cell invasion, EMT, and the TAM differentiation of macrophages [14, 15]. Moreover, these effects were suppressed by the loss of HSP90 in sEVs [15]. Our present observations that ATP7B-high CDDP-resistant HNSC cells secreted HSP90- and EpCAM-rich sEVs and promoted the EMT are consistent with these reports (Figs. 2E, F and 4D, Supplementary Fig. S1C–F).

Our present results demonstrated that sEV transmission derived from CDDP-resistant cells weakens CDDP sensitivity by enhancing ATP7B in recipient cells (Fig. 4F–H). The sEV-induced enhancement of copper transporter activity that we verified in this study (Fig. 4F–H) is a novel finding. However, the results of this study did not clarify the mechanism by which sEV-specific factors directly upregulate intracellular ATP7B. A comprehensive transcriptomic analysis of sEVs derived from CDDP-resistant HNSC cells may enable the identification of factors that upregulate ATP7B.

In recent years, the importance of applying proteomics and metabolomics to EV research has been gaining attention in efforts to elucidate the mechanisms of specific diseases [14, 40–42]. The composition of sEVs reflects the pathological and physiological state of the cells of origin. To narrow down the number of potential sEV cargo candidates that drive ATP7B elevation, it is necessary to conduct follow-up transcriptomic analyses such as proteomics and metabolomics. In the latest sEV research, proteomics using mass spectrometry was performed on sEV derived from cancer cells lacking Syntenin, a factor that regulates sEV biogenesis via endosomes [43]. Differences in patient-derived serum sEV protein profiles according to disease status may have potential significance for cancer diagnosis, prognosis, and treatment [44]. It will be essential to perform sEV omics as the next task to clarify CDDP resistance mediated by sEV and the mechanism of sEV biogenesis by ATP7B in our research as well.

GW4869 is a cell-permeable selective compound that acts as a potent non-competitive inhibitor of the membrane enzyme nSMase, thereby inhibiting the generation of EVs [26]. The suspected mechanism of GW4869 is its inhibition of EV generation by blocking the maturation pathway from early endosomes to late endosomes [26, 45]. This is consistent with our present finding that GW4869 weakened Rab7 but left Rab5 unchanged (Fig. 5B). Together, our previous investigation [8] and present findings demonstrate that GW4869 enhances the cytotoxic effect of CDDP by attenuating ATP7B; in addition, the copper chaperone Atox1 has also been shown to mediate CDDP resistance through a different mechanism [46]. There are reports that Atox1 deficiency causes a loss of the ability of CDDP to be transported to ATP7A and ATP7B [47, 48]. An increased expression of Atox1 is associated with CDDP resistance in multiple tumor cell lines [49]. Consistent with these concepts, we observed that GW4869 attenuates Atox1, thereby enhancing CDDP responsiveness (Fig. 5A–C). An inhibition of Atox1 may be involved in the CDDP trapping/efflux by reducing the supply of CDDP to ATP7B [50, 51].

In addition to a 2D culture, we used a 3D culture and an in vivo investigation as a test for GW4869 (Figs. 6 and 7). Three-dimensional culture models are useful as a test prior to animal testing in drug evaluation tests [22, 23]. As shown in Fig. 6A, B, the combination of CDDP and GW4869 enhanced the cytotoxic effect by reducing the hypoxic regions and increasing the detection of cleaved caspase 3 in HNSC spheroids; a spheroid tissue evaluation is also very effective as a future strategy for investigating changes in ATP7B localization [22, 23]. GW4869 did not affect the in vivo tumor growth but caused a reduction in the ATP7B expression in tumor tissues (Fig. 7A, B, Supplementary Fig. S4A, B). Moreover, the western blotting of serum-derived sEV fractions revealed that GW4869 clearly attenuated sEV proteins (Fig. 7D, E). Dinkins et al. demonstrated a decrease in the protein concentration and a reduction in the levels of ALG-2-interacting protein X (Alix) in sEVs isolated from the serum of mice that received daily intraperitoneal injections of GW4869 [52]. In addition, Essandoh et al. demonstrated that the administration of GW4869 at a dose of 2.5 μg/g body weight to mice resulted in a 33% reduction in serum sEV levels [53].

In terms of the combined effect with antitumor drugs, strong antitumor responses have been observed when GW4869 was administered together with bortezomib in a murine model of multiple myeloma [54]. GW4869 has also been shown to enhance the sensitivity of gefitinib by suppressing the expression of EV-HSP90α and EMT in non-small cell lung cancer [55]. Our validation showed that GW4869 did not induce cell death or tumor shrinking in HNSC cells in either 2D or 3D cultures, nor in animal models (Figs. 6 and 7) [8], suggesting that expecting antitumor effects from this compound alone may be difficult. Further developments of our present study could be achieved if it is observed that CDDP and GW4869 exert more effective antitumor effects in vivo. GW4869 may play an important role in the clinical treatment of CDDP-resistant HNSC, but this needs to be confirmed by further experimental and clinical studies.

Our present findings may lead to the identification of new molecular targets that could be used for more effective treatments of CDDP-resistant HNSC. We plan to conduct further research and collect relevant clinical data to substantiate our experimental results.

## Supplementary Material

Supplementary table 3

Supplementary table 2

Supplementary table 1

Supplementary figures

**Supplementary information** The online version contains supplementary material available at https://doi.org/10.1038/s41417-025-00975-9.

## Data Availability

The datasets generated and/or analyzed in this study are available from the corresponding author on reasonable request.

## References

[1] Hu H, Li B, Wang J, Tan Y, Xu M, Xu W, New advances into cisplatin resistance in head and neck squamous carcinoma: mechanisms and therapeutic aspects. Biomed Pharmacother. 2023;163:114778. 37137185 10.1016/j.biopha.2023.114778

[2] Dorna D, Paluszczak J. Targeting cancer stem cells as a strategy for reducing chemotherapy resistance in head and neck cancers. J Cancer Res Clin Oncol. 2023;149:13417–35. 37453969 10.1007/s00432-023-05136-9 PMC10587253

[3] Kanno Y, Chen CY, Lee HL, Chiou JF, Chen YJ. Molecular mechanisms of chemotherapy resistance in head and neck cancers. Front Oncol. 2021;11:640392. 34026617 10.3389/fonc.2021.640392 PMC8138159

[4] Bompiani KM, Tsai CY, Achatz FP, Liebig JK, Howell SB. Copper transporters and chaperones CTR1, CTR2, ATOX1, and CCS as determinants of cisplatin sensitivity. Metallomics. 2016;8:951–62. 27157188 10.1039/c6mt00076b PMC5025354

[5] Ito F, Kato K, Yanatori I, Maeda Y, Murohara T, Toyokuni S. Matrigel-based organoid culture of malignant mesothelioma reproduces cisplatin sensitivity through CTR1. BMC Cancer. 2023;23:487. 37254056 10.1186/s12885-023-10966-4 PMC10230733

[6] Spreckelmeyer S, van der Zee M, Bertrand B, Bodio E, Stürup S, Casini A. Relevance of copper and organic cation transporters in the activity and transport mechanisms of an anticancer cyclometallated gold(III) compound in comparison to cisplatin. Front Chem. 2018;6:377. 30234099 10.3389/fchem.2018.00377 PMC6131305

[7] Marjamaa A, Gibbs B, Kotrba C, Masamha CP. The role and impact of alternative polyadenylation and miRNA regulation on the expression of the multidrug resistance-associated protein 1 (MRP-1/ABCC1) in epithelial ovarian cancer. Sci Rep. 2023;13:17476. 37838788 10.1038/s41598-023-44548-y PMC10576765

[8] Ogawa T, Ono K, Ryumon S, Kawai H, Nakamura T, Umemori K, Novel mechanism of cisplatin resistance in head and neck squamous cell carcinoma involving extracellular vesicles and a copper transporter system. Head Neck. 2024;46:636–50. 38164660 10.1002/hed.27620

[9] Ryumon S, Okui T, Kunisada Y, Kishimoto K, Shimo T, Hasegawa K, Ammonium tetrathiomolybdate enhances the antitumor effect of cisplatin via the suppression of ATPase copper transporting beta in head and neck squamous cell carcinoma. Oncol Rep. 2019;42:2611–21. 31638244 10.3892/or.2019.7367 PMC6826331

[10] Guo Z, Orädd F, Bågenholm V, Grønberg C, Ma JF, Ott P, Diverse roles of the metal binding domains and transport mechanism of copper transporting P-type ATPases. Nat Commun. 2024;15:2690. 38538615 10.1038/s41467-024-47001-4 PMC10973460

[11] Tsvetkov P, Coy S, Petrova B, Dreishpoon M, Verma A, Abdusamad M, Copper induces cell death by targeting lipoylated TCA cycle proteins. Science. 2022;375:1254–61. 35298263 10.1126/science.abf0529 PMC9273333

[12] Fang T, Chen W, Sheng Y, Yuan S, Tang Q, Li G, Tetrathiomolybdate induces dimerization of the metal-binding domain of ATPase and inhibits platination of the protein. Nat Commun. 2019;10:186. 30643139 10.1038/s41467-018-08102-z PMC6331642

[13] Kumar MA, Baba SK, Sadida HQ, Marzooqi SA, Jerobin J, Altemani FH, Extracellular vesicles as tools and targets in therapy for diseases. Signal Transduct Target Ther. 2024;9:27. 38311623 10.1038/s41392-024-01735-1 PMC10838959

[14] Ono K, Eguchi T, Sogawa C, Calderwood SK, Futagawa J, Kasai T, HSP-enriched properties of extracellular vesicles involve survival of metastatic oral cancer cells. J Cell Biochem. 2018;119:7350–62. 29768689 10.1002/jcb.27039

[15] Ono K, Sogawa C, Kawai H, Tran MT, Taha EA, Lu Y, Triple knockdown of CDC37, HSP90-alpha and HSP90-beta diminishes extracellular vesicles-driven malignancy events and macrophage M2 polarization in oral cancer. J Extracell Vesicles. 2020;9:1769373. 33144925 10.1080/20013078.2020.1769373 PMC7580842

[16] Liu K, Gao L, Ma X, Huang JJ, Chen J, Zeng L, Long non-coding RNAs regulate drug resistance in cancer. Mol Cancer. 2020;19:54. 32164712 10.1186/s12943-020-01162-0 PMC7066752

[17] Hwang H, Kim J, Kim TH, Han Y, Choi D, Cho S, Exosomal miR-6126 as a novel therapeutic target for overcoming resistance of anti-cancer effect in hepatocellular carcinoma. BMC Cancer. 2024;24:1557. 39702014 10.1186/s12885-024-13342-y PMC11660897

[18] Qin C, Zhao B, Wang Y, Li Z, Li T, Zhao Y, Extracellular vesicles miR-31–5p promotes pancreatic cancer chemoresistance via regulating LATS2-Hippo pathway and promoting SPARC secretion from pancreatic stellate cells. J Extracell Vesicles. 2024;13:e12488. 39104296 10.1002/jev2.12488 PMC11300957

[19] Zhao S, Pan T, Deng J, Cao L, Vicencio JM, Liu J, Exosomal transfer of miR-181b-5p confers senescence-mediated doxorubicin resistance via modulating BCLAF1 in breast cancer. Br J Cancer. 2023;128:665–77. 36522479 10.1038/s41416-022-02077-x PMC9938221

[20] Fujiwara T, Eguchi T, Sogawa C, Ono K, Murakami J, Ibaragi S, Anti-EGFR antibody cetuximab is secreted by oral squamous cell carcinoma and alters EGF-driven mesenchymal transition. Biochem Biophys Res Commun. 2018;503:1267–72. 30017201 10.1016/j.bbrc.2018.07.035

[21] Lukanović D, Herzog M, Kobal B, Černe K. The contribution of copper efflux transporters ATP7A and ATP7B to chemoresistance and personalized medicine in ovarian cancer. Biomed Pharmacother. 2020;129:110401. 32570116 10.1016/j.biopha.2020.110401

[22] Umemori K, Ono K, Eguchi T, Kawai H, Nakamura T, Ogawa T, EpEX, the soluble extracellular domain of EpCAM, resists cetuximab treatment of EGFR-high head and neck squamous cell carcinoma. Oral Oncol. 2023;142:106433. 37236125 10.1016/j.oraloncology.2023.106433

[23] Ono K, Sato K, Nakamura T, Yoshida Y, Murata S, Yoshida K, Reproduction of the antitumor effect of cisplatin and cetuximab using a three-dimensional spheroid model in oral cancer. Int J Med Sci. 2022;19:1320–33. 35928727 10.7150/ijms.74109 PMC9346383

[24] Borchers AC, Langemeyer L, Ungermann C. Who’s in control? Principles of Rab GTPase activation in endolysosomal membrane trafficking and beyond. J Cell Biol. 2021;220:e202105120. 10.1083/jcb.202105120 PMC8366711 34383013

[25] Parisi S, Polishchuk EV, Allocca S, Ciano M, Musto A, Gallo M, Characterization of the most frequent ATP7B mutation causing Wilson disease in hepatocytes from patient induced pluripotent stem cells. Sci Rep. 2018;8:6247. 29674751 10.1038/s41598-018-24717-0 PMC5908878

[26] Kim JH, Lee CH, Baek MC. Dissecting exosome inhibitors: therapeutic insights into small-molecule chemicals against cancer. Exp Mol Med. 2022;54:1833–43. 36446847 10.1038/s12276-022-00898-7 PMC9707221

[27] Kuo MT, Huang YF, Chou CY, Chen HHW. Targeting the copper transport system to improve treatment efficacies of platinum-containing drugs in cancer chemotherapy. Pharmaceuticals. 2021;14:549. 34201235 10.3390/ph14060549 PMC8227247

[28] Yanagimoto C, Harada M, Kumemura H, Abe M, Koga H, Sakata M, Copper incorporation into ceruloplasmin is regulated by Niemann-Pick C1 protein. Hepatol Res. 2011;41:484–91. 21518405 10.1111/j.1872-034X.2011.00788.x

[29] Hartwig C, Méndez GM, Bhattacharjee S, Vrailas-Mortimer AD, Zlatic SA, Freeman AAH, Golgi-dependent copper homeostasis sustains synaptic development and mitochondrial content. J Neurosci. 2021;41:215–33. 33208468 10.1523/JNEUROSCI.1284-20.2020 PMC7810662

[30] Safaei R, Adams PL, Mathews RA, Manorek G, Howell SB. The role of metal binding and phosphorylation domains in the regulation of cisplatin-induced trafficking of ATP7B. Metallomics. 2013;5:964–72. 23803742 10.1039/c3mt00131h PMC4030745

[31] Gupta A, Bhattacharjee A, Dmitriev OY, Nokhrin S, Braiterman L, Hubbard AL, Cellular copper levels determine the phenotype of the Arg875 variant of ATP7B/Wilson disease protein. Proc Natl Acad Sci USA. 2011;108:5390–5. 21406592 10.1073/pnas.1014959108 PMC3069211

[32] van den Berghe PV, Stapelbroek JM, Krieger E, de Bie P, van de Graaf SF, de Groot RE, Reduced expression of ATP7B affected by Wilson disease-causing mutations is rescued by pharmacological folding chaperones 4-phenylbutyrate and curcumin. Hepatology. 2009;50:1783–95. 19937698 10.1002/hep.23209

[33] Guo Y, Nyasae L, Braiterman LT, Hubbard AL. NH2-terminal signals in ATP7B Cu-ATPase mediate its Cu-dependent anterograde traffic in polarized hepatic cells. Am J Physiol Gastrointest Liver Physiol. 2005;289:G904–16. 15994426 10.1152/ajpgi.00262.2005

[34] Zhitomirsky B, Assaraf YG. Lysosomes as mediators of drug resistance in cancer. Drug Resist Updat. 2016;24:23–33. 26830313 10.1016/j.drup.2015.11.004

[35] Su H, Yang F, Fu R, Li X, French R, Mose E, Cancer cells escape autophagy inhibition via NRF2-induced macropinocytosis. Cancer Cell. 2021;39:678–693.e11. 33740421 10.1016/j.ccell.2021.02.016 PMC8119368

[36] Zhou G, Gu Y, Zhu Z, Zhang H, Liu W, Xu B, Exosome mediated cytosolic cisplatin delivery through clathrin-independent endocytosis and enhanced anti-cancer effect via avoiding endosome trapping in cisplatin-resistant ovarian cancer. Front Med. 2022;9:810761. 10.3389/fmed.2022.810761 PMC9113028 35592860

[37] Harada M, Sakisaka S, Kawaguchi T, Kimura R, Taniguchi E, Koga H, Copper does not alter the intracellular distribution of ATP7B, a copper-transporting ATPase. Biochem Biophys Res Commun. 2000;275:871–6. 10973814 10.1006/bbrc.2000.3403

[38] Weiss KH, Lozoya JC, Tuma S, Gotthardt D, Reichert J, Ehehalt R, Copper-induced translocation of the Wilson disease protein ATP7B independent of Murr1/COMMD1 and Rab7. Am J Pathol. 2008;173:1783–94. 18974300 10.2353/ajpath.2008.071134 PMC2626389

[39] Safaei R, Larson BJ, Cheng TC, Gibson MA, Otani S, Naerdemann W, Abnormal lysosomal trafficking and enhanced exosomal export of cisplatin in drug-resistant human ovarian carcinoma cells. Mol Cancer Ther. 2005;4:1595–604. 16227410 10.1158/1535-7163.MCT-05-0102

[40] Du Y, Qiu R, Chen L, Chen Y, Zhong Z, Li P, Identification of serum exosomal metabolomic and proteomic profiles for remote ischemic preconditioning. J Transl Med. 2023;21:241. 37009888 10.1186/s12967-023-04070-1 PMC10069038

[41] Ono K, Eguchi T. Proteomic profiling of the extracellular vesicle chaperone in cancer. Methods Mol Biol. 2023;2693:233–49. 37540439 10.1007/978-1-0716-3342-7_18

[42] Eguchi T, Okusha Y, Lu Y, Ono K, Taha EA, Fukuoka S. Comprehensive method for exosome isolation and proteome analysis for detection of CCN factors in/on exosomes. Methods Mol Biol. 2023;2582:59–76. 36370344 10.1007/978-1-0716-2744-0_6

[43] Irmer B, Angenendt A, Camoin L, Audebert S, Geyer C, Gerwing M, Syntenin controls extracellular vesicle-induced tumour migration by regulating the expression of adhesion proteins on small extracellular vesicles. J Extracell Vesicles. 2025;14:e70133. 40831280 10.1002/jev2.70133 PMC12365386

[44] Cao Z, Wang Y, Wu J, Tang X, Qian Z, Zhang Z, Serum small extracellular vesicles-derived BST2 as a biomarker for papillary thyroid microcarcinoma promotes lymph node metastasis. Cancer Gene Ther. 2025;32:38–50. 39558134 10.1038/s41417-024-00854-9 PMC11772248

[45] Peng W, Chen S, Ma J, Wei W, Lin N, Xing J, Endosomal trafficking participates in lipid droplet catabolism to maintain lipid homeostasis. Nat Commun. 2025;16:1917. 39994216 10.1038/s41467-025-57038-8 PMC11850777

[46] Petruzzelli R, Polishchuk RS. Activity and trafficking of copper-transporting ATPases in tumor development and defense against platinum-based drugs. Cells. 2019;8:1080. 31540259 10.3390/cells8091080 PMC6769697

[47] Hua H, Günther V, Georgiev O, Schaffner W. Distorted copper homeostasis with decreased sensitivity to cisplatin upon chaperone Atox1 deletion in Drosophila. Biometals. 2011;24:445–53. 21465178 10.1007/s10534-011-9438-1

[48] Safaei R, Maktabi MH, Blair BG, Larson CA, Howell SB. Effects of the loss of Atox1 on the cellular pharmacology of cisplatin. J Inorg Biochem. 2009;103:333–41. 19124158 10.1016/j.jinorgbio.2008.11.012 PMC2919289

[49] Palm-Espling ME, Lundin C, Björn E, Naredi P, Wittung-Stafshede P. Interaction between the anticancer drug Cisplatin and the copper chaperone Atox1 in human melanoma cells. Protein Pept Lett. 2014;21:63–8. 23988033 10.2174/09298665113209990036

[50] Mariniello M, Petruzzelli R, Wanderlingh LG, La Montagna R, Carissimo A, Pane F, Synthetic lethality screening identifies FDA-approved drugs that overcome ATP7B-mediated tolerance of tumor cells to cisplatin. Cancers. 2020;12:608. 32155756 10.3390/cancers12030608 PMC7139527

[51] Zhao L, Cheng Q, Wang Z, Xi Z, Xu D, Liu Y. Cisplatin binds to human copper chaperone Cox17: the mechanistic implication of drug delivery to mitochondria. Chem Commun. 2014;50:2667–9. 10.1039/c3cc48847k 24473407

[52] Dinkins MB, Dasgupta S, Wang G, Zhu G, Bieberich E. Exosome reduction in vivo is associated with lower amyloid plaque load in the 5XFAD mouse model of Alzheimer’s disease. Neurobiol Aging. 2014;35:1792–800. 24650793 10.1016/j.neurobiolaging.2014.02.012 PMC4035236

[53] Essandoh K, Yang L, Wang X, Huang W, Qin D, Hao J, Blockade of exosome generation with GW4869 dampens the sepsis-induced inflammation and cardiac dysfunction. Biochim Biophys Acta. 2015;1852:2362–71. 26300484 10.1016/j.bbadis.2015.08.010 PMC4581992

[54] Faict S, Muller J, De Veirman K, De Bruyne E, Maes K, Vrancken L, Exosomes play a role in multiple myeloma bone disease and tumor development by targeting osteoclasts and osteoblasts. Blood Cancer J. 2018;8:105. 30409995 10.1038/s41408-018-0139-7 PMC6224554

[55] Wan X, Fang Y, Du J, Cai S, Dong H. GW4869 can inhibit epithelial-mesenchymal transition and extracellular HSP90α in gefitinib-sensitive NSCLC cells. Onco-Targets Ther. 2023;16:913–22. 38021444 10.2147/OTT.S428707 PMC10640835

