# Supplementary material for: ATPase copper transporting beta contributes to cisplatin resistance as a regulatory factor of extracellular vesicles in head and neck squamous cell carcinoma
Source: Cancer Gene Ther. 2025 Oct 17;33(1):39–54. doi: 10.1038/s41417-025-00975-9 (PMC12783013; doi:10.1038/s41417-025-00975-9)
Supplement: Supplementary file 1 — Supplementary figures [file 41417_2025_975_MOESM1_ESM.docx]

**SUPPLEMENTARY MATERIALS**

Supplementary Figures S1–S4

Supplementary Tables S1–S3


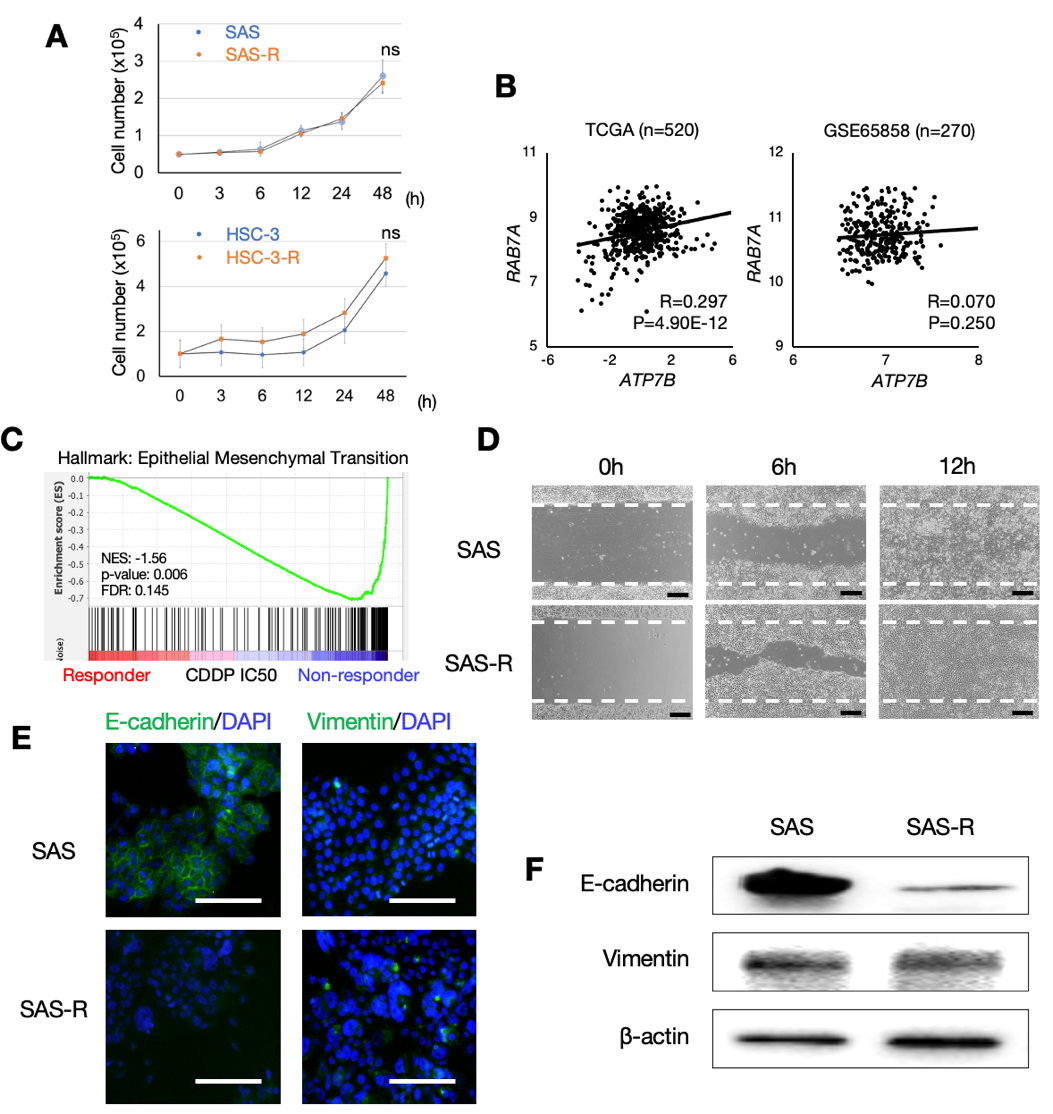


**Suppl. Fig. S1. (A)** Cell proliferation curves comparing the parental cells and the CDDP-resistant cells in each cell line (n=3). Error bars: standard deviation (SD). Student t test was used for the comparison. ns: not significant. **(B)** Scatterplots showing the correlations between the expressions of *ATP7B* and *RAB7A* in the HNSC clinical samples based on the TCGA-HNSC cohort (*left*, n=520) and the GSE65858 dataset (*right*, n=270). **(C)** The GSEA results showing the hallmark signature from MSigDB by comparing the CDDP responders and non-responders of the HNSC cell lines in the GDSC database. A set of genes related to the epithelial mesenchymal transition (EMT) was significantly enriched in the CDDP non-responder cell lines. NES: normalization enrichment score, FDR: false discovery rate. **(D)** Wound healing assay comparing the cell migration ability of the parent and CDDP-resistant SAS cells. The *dotted line* indicates the boundary immediately after scratching (i.e., 0 hr). Scale bar: 200 μm. **(E)** ICC showing the E-cadherin or vimentin protein expression in the parent and CDDP-resistant SAS cells. Slides were incubated with anti-E-cadherin (#24E10, #3195S, Cell Signaling Technology, dilution 1:1 000), or anti-vimentin (#D21H3, #5741S, Cell Signaling Technology, dilution 1:100) antibodies overnight at 4°C and with anti-rabbit IgG AlexaFluor488 (#A-11008, ThermoFisher Scientific, dilution 1:500) for 60 min. Slides were stained with nuclear stain DAPI. E-cadherin and vimentin are shown in *green* and DAPI in *blue*. Scale bar: 200 μm. **(F)** Western blotting showing E-cadherin and vimentin in the parent and CDDP-resistant SAS cells. Membrane was blocked in 5% skim milk in Tris-buffered saline containing 0.05% Tween-20 for 60 min, incubated with primary antibodies (dilution 1:1 000), and then incubated with HRP-conjugated secondary antibodies (dilution 1:5 000). Blots were visualized using a Clarity ECL substrate and a ChemiDoc MP system (Bio-Rad). β-Actin = loading control.

**
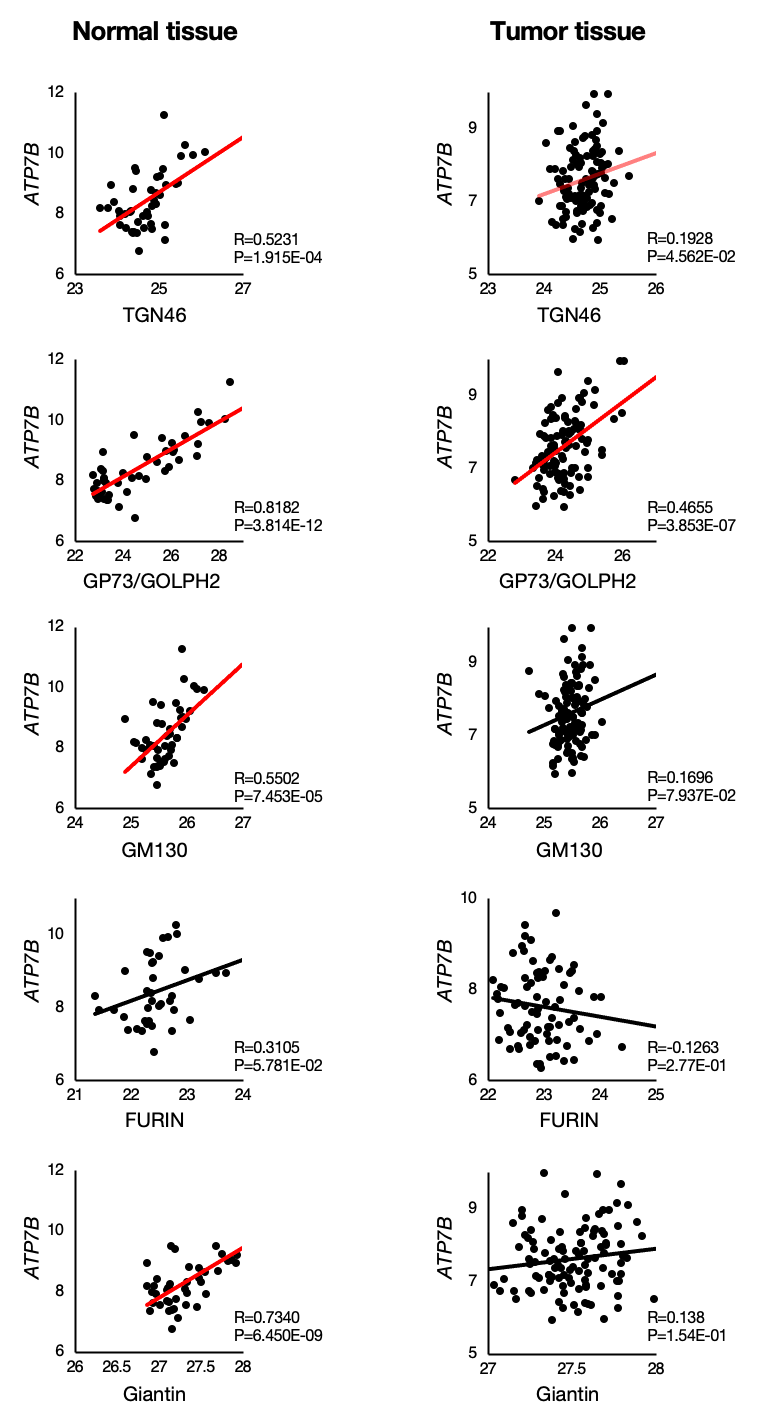
**

**Suppl. Fig. S2.** Scatterplots showing the correlations between the expression of ATP7B and the expressions of the Golgi apparatus markers TGN46, GP73/GOLPH2, GM130, FURIN, and Giantin in the normal tissues and tumor tissues based on the CPTAC-HNSC cohort.

**
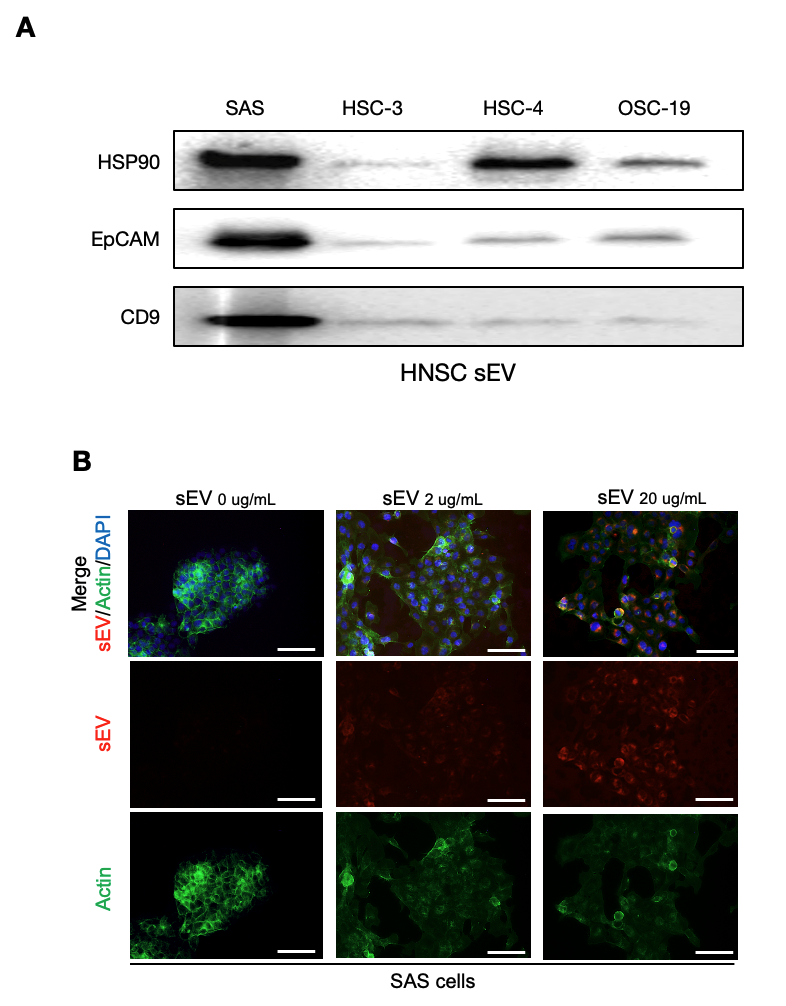
**

**Suppl. Fig. S3. (A)** Western blotting of HSP90, EpCAM, and CD9 in sEVs derived from four different HNSC cell lines. Membrane was blocked in 5% skim milk in Tris-buffered saline containing 0.05% Tween-20 for 60 min, incubated with primary antibodies (dilution 1:1 000), and then incubated with HRP-conjugated secondary antibodies (dilution 1:5 000). Blots were visualized using a Clarity ECL substrate and a ChemiDoc MP system (Bio-Rad). **(B)** ICC showing fluorescently labeled sEVs after the transmission of sEVs at various concentrations into recipient cells for 24 hr. An ExoGlow-protein EV labeling kit (System Biosciences) was used and Isolated sEVs were incubated for 20 min with the labeling dye (1:500 in PBS) at 37°C to induce vesicular protein conjugation with the dye molecules. The solution was then treated with ExoQuick-TC solution and incubated for 2 hr at 4°C, followed by centrifugation at 10 000g for 10 min to remove unlabeled reagent molecules. For a negative control, sterile PBS was incubated with labeling dye and treated in the same manner. Labeled sEV pellets were dissolved in PBS. The labeled sEVs were added to the culture medium of SAS cells in 96-well plates at 0 μg/mL, 2 μg/mL or 20 μg/mL. The cells were fixed with 4% paraformaldehyde in PBS for 10 min and permeabilized with 0.5% Tween-20 in PBS for 5 min. The cells were incubated with ActinGreen488 (ThermoFisher) for 30 min and with DAPI for 5 min. Fluorescence images were taken using a BZ-X700 microscope (Keyence). sEV is shown in *red*, actin in *green*, and DAPI in *blue*. EV-free PBS was used as a control. Scale bar: 100 μm.


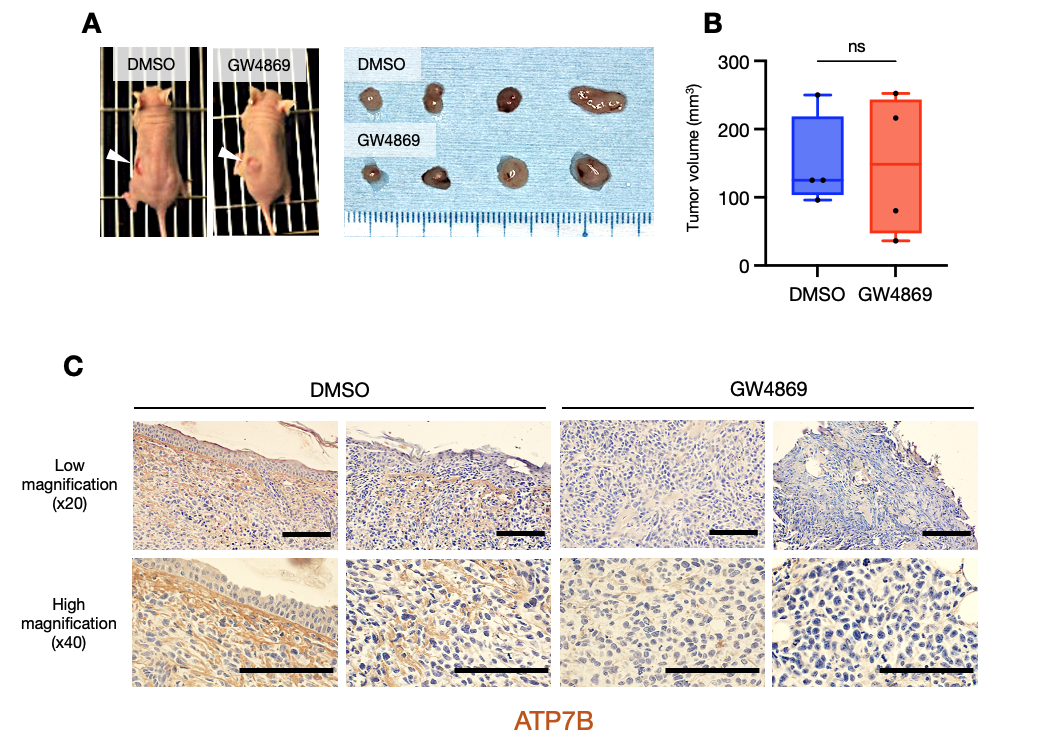


**Suppl. Fig. S4. (A)** Photographs of representative mice just before sacrifice and the excised tumors treated with or without GW4869 (n=4). The tumors were located in the left dorsal subcutaneous tissue (arrowheads). Scale bar: 10 mm. **(B)** The longest diameters of the excised tumors treated with or without GW4869 (n=4). The lines indicate the mean and range. Welch's t test was used for the comparison. ns: not significant. **(C)** Immunohistochemical staining images of ATP7B in tumor tissues excised from mice other than those shown in Figure 6C. The upper and lower images were provided from the same tumor, the *upper row* showing low-magnification images, and the *lower row* showing high-magnification images. The immunohistochemistry (IHC) examination was performed using the anti-ATP7B antibody (#NB100-360, Novus Biologicals). Scale bar: 100 μm.

**Supplementary Table Captions**

**Suppl. Table S1.** GO and KEGG pathway terms of the 11 ATP7B-related proteins detected in the PPI databases (See the provided Excel file)

**Suppl. Table S2.** Subcellular localization terms of the top 50 proteins that were positively correlated with ATP7B in normal tissues based on the GO-CC and the Jensen Compartment database (See the provided Excel file)

**Suppl. Table S3.** Subcellular localization terms of the top 50 proteins that were positively correlated with ATP7B in tumor tissues based on the GO-CC and the Jensen Compartment database (See the provided Excel file)
